# BMP-Mediated Functional Cooperation between *Dlx5;Dlx6* and *Msx1;Msx2* during Mammalian Limb Development

**DOI:** 10.1371/journal.pone.0051700

**Published:** 2013-01-29

**Authors:** Maxence Vieux-Rochas, Kamal Bouhali, Stefano Mantero, Giulia Garaffo, Paolo Provero, Simonetta Astigiano, Ottavia Barbieri, Mariano F. Caratozzolo, Apollonia Tullo, Luisa Guerrini, Yvan Lallemand, Benoît Robert, Giovanni Levi, Giorgio R. Merlo

**Affiliations:** 1 Evolution des Régulations Endocriniennes, Centre national de la recherche scientifique, UMR-7221, Muséum National d’Histoire Naturelle, Paris, France; 2 Molecular Biotechnology Center, University of Torino, Torino, Italy; 3 Istituto Di Ricovero e Cura a Carattere Scientifico Azienda Ospedale Università San Martino, IST Istituto Nazionale per la Ricerca sul Cancro, Genova, Italy; 4 Department of Experimental Medicine, University of Genova, Genova, Italy; 5 Institute for Biomedical Technologies, National Research Council, Bari, Italy; 6 Department of Biosciences, University of Milano, Milano, Italy; 7 Institut Pasteur, Department of Developmental Biology, Centre national de la recherche scientifique URA-2578, Paris, France; 8 Dulbecco Telethon Institute, University of Torino, Torino, Italy; Ecole Normale Supérieure de Lyon, France

## Abstract

The Dlx and Msx homeodomain transcription factors play important roles in the control of limb development. The combined disruption of *Msx1* and *Msx2*, as well as that of *Dlx5* and *Dlx6,* lead to limb patterning defects with anomalies in digit number and shape. *Msx1;Msx2* double mutants are characterized by the loss of derivatives of the anterior limb mesoderm which is not observed in either of the simple mutants. *Dlx5;Dlx6* double mutants exhibit hindlimb ectrodactyly. While the morphogenetic action of *Msx* genes seems to involve the BMP molecules, the mode of action of *Dlx* genes still remains elusive. Here, examining the limb phenotypes of combined *Dlx* and *Msx* mutants we reveal a new Dlx-Msx regulatory loop directly involving BMPs. In *Msx1;Dlx5;Dlx6* triple mutant mice (TKO), beside the expected ectrodactyly, we also observe the hallmark morphological anomalies of *Msx1;Msx2* double mutants suggesting an epistatic role of *Dlx5* and *Dlx6* over *Msx2*. In *Msx2;Dlx5;Dlx6* TKO mice we only observe an aggravation of the ectrodactyly defect without changes in the number of the individual components of the limb. Using a combination of qPCR, ChIP and bioinformatic analyses, we identify two Dlx/Msx regulatory pathways: 1) in the anterior limb mesoderm a non-cell autonomous Msx-Dlx regulatory loop involves BMP molecules through the AER and 2) in AER cells and, at later stages, in the limb mesoderm the regulation of *Msx2* by *Dlx5* and *Dlx6* occurs also cell autonomously. These data bring new elements to decipher the complex AER-mesoderm dialogue that takes place during limb development and provide clues to understanding the etiology of congenital limb malformations.

## Introduction

The developing vertebrate limb is widely adopted as a model to study cell-cell signaling, pattern formation and morphogenesis, and has provided a wealth of knowledge of the function and regulation of specific transcription factors and signaling molecules [1], [2], [3]. The phenotype of a large set of mutant mice with limb defects led to the identification of genes and regulatory pathways essential for normal limb development. The spatio-temporal organization of the complex network of signaling and transcriptional regulations has been elucidated only in part. In brief, genes and regulatory modules can be related to the activation/maintenance/regression of three signaling systems: a) Sonic hedgehog (SHH) and the Zone of Polarizing Activity (ZPA), for the control of digit patterning along the antero-posterior axis [4], [5], [6], b) Fibroblast Growth Factors (FGFs) and the Apical Ectodermal Ridge (AER), for the control of proximo-distal growth and for ZPA maintenance [7], [8], [9], [10], [11], and c) Lmx1B, in the mesoderm, and Wnt7a and En-1, in the ectoderm, for dorso-ventral specification [12], [13], [14], [15], [16]. The three signaling systems are organized in precise time- and space-restricted manners, and are integrated in self-regulatory modules that assure the acquisition or the correct digit complements, limb morphogenesis and overall growth [4], [17], [18], [19]. The signaling molecules of the Bone Morphogenetic Protein (BMP) class have been proposed to link these three systems together [20], [21], [22], [23], [24], [25], thus they are regarded as key players in the coordination of limb patterning, morphogenesis and growth along the three axes.

Proximo-distal limb development and digit extension is directly controlled by the signaling activity of the AER via expression of morphogens of the FGF family [7], [8], [10], [26] and their regulation by other signaling molecules such as SHH and BMP antagonists [20], [21], [27], [28]. In this scenario, the *Dlx* and *Msx* homeobox transcription factors, expressed in the AER and the mesoderm of the limb buds, play an important morphogenetic role, although their functions and regulations are yet to be defined at the molecular level. *Dlx5;Dlx6* double knock-out (DKO) mice are characterized by loss of the central digit(s) and/or fusion with the lateral ones [29], [30]; these mice constitute a model of the human congenital defect ectrodactyly or Split Hand Foot Malformation type I, a condition linked to genomic alterations encompassing the *DLX5;DLX6* region, and for which point mutations in the DNA-binding domain of *DLX5* have recently been found [31]. The double inactivation of *Msx1* and *Msx2* genes results in moderate-penetrance polydactyly of the forelimbs (FL) and oligodactyly of the hindlimbs (HL) (loss of the anterior digits and part of the tibia in the zeugopod) [32], [33], [34].

Studies on the cellular and molecular functions of Dlx and Msx proteins during limb development have met with difficulties owing to several reasons. First, none of the single knockout for *Dlx5, Dlx6, Msx1* or *Msx2* shows evident limb defects [29], [32], [35], [36], [37], [38], [39], [40], [41], suggesting some degree of functional redundancy. Second, members of the Dlx and Msx families are expressed both in the AER and in restricted regions of the limb mesoderm [42], [43]. Third, *in vitro* the Dlx and Msx proteins compete for the same DNA binding sites, form heterodimers *via* their homeodomain and reciprocally inhibit their transcriptional activities [44], due to the high degree of homology of their homeodomains [37], [43], [45], [46], [47]. However, current literature suggest that Dlx and Msx proteins have distinct functions: Msx1 and Msx2 are known to control cell proliferation and differentiation in a variety of cell types [48], [49], [50], [51], while *Dlx* genes are implicated in the differentiation of specific cell lineages, such as forebrain interneurons [52], [53], [54], olfactory receptor neurons [55], osteoblasts [35], [56], and the AER and ectoderm [29], [30], [57], [58]. Notably, *Dlx* and *Msx* genes have been shown to cooperate only in specific cases [59], [60], [61], but not at all sites where they are co-expressed. However, Dlx5 has been shown to regulate *Msx2* transcription in the AER, via homeodomain binding elements present in the gene’s promoter [62], [63].

We investigate possible interactions between *Dlx5;Dlx6* and *Msx1* or *Msx2* in limb development, by generating double and triple *Dlx;Msx* compound mutant mice and analyzing their limb phenotypes. The limb phenotype of *Msx1;Dlx5;Dlx6* triple knock-out (TKO) mice shows features of the *Msx1;Msx2* DKO phenotype [32], leading to the conclusion that *Dlx5;Dlx6* control the expression of *Msx2*, but not of *Msx1*. In contrast, the limb phenotype of *Msx2;Dlx5;Dlx6* TKO mice is consistent with a severe aggravation of the ectrodactyly defect. We also re-examine the spatio-temporal expression of *Dlx* and *Msx* genes in FLs and HLs and observe that in the AER these genes are co-expressed whereas in the anterior mesenchyme *Msx* expression precedes that of *Dlx*, ruling out a direct regulation of Msx by Dlx in this territory. Combining these findings with qPCR expression analyses on the limb buds of different compound embryos, and with ChIP data, we propose that two modes of regulation coexist during limb development: 1) a direct transcriptional regulation of *Msx2 by* Dlx proteins in the AER and, later on, in the limb mesoderm, and 2) a Bmp2- and Bmp4-mediated non-cell autonomous regulatory loop between the AER and the anterior limb mesoderm.

## Materials and Methods

### Mouse Strains and Breeding

All animal procedures were reviewed and approved by the Ethical Committee of the University of Torino and University of Genova, the Italian Ministry of Health and the French Ministère de l’Enseignement supérieur et de la Recherche. No surgery or other manipulation on adult animals was used in this study. All efforts were made to minimize suffering. Generation and genotyping of the *Dlx5^lacZ/+^* (hereafter named *Dlx5* ^+/−^); *Dlx5;Dlx6^neo/+^* (hereafter named *Dlx5;Dlx6* ^+/−^)*; Msx1^lacZ/+^* and *Msx2^lacZ/+^* (hereafter named, respectively, *Msx1* ^+/−^ and *Msx2* ^+/−^) have been previously reported [29], [32], [35], [39]. These mutations were maintained on a C57B6;DBA F1 mixed genetic background, throughout. TKO embryos and newborn were obtained by crossbreeding either the *Msx1^+/−^* or the *Msx2^+/−^* single heterozygous parents with the *Dlx5;Dlx6^+/−^* double heterozygous ones, and then crossing the triple heterozygotes. Following mating, the day of the vaginal plug was considered as embryonic age 0.5 (E0.5).

### Skeletal Preparation and β-galactosidase Detection

Cartilage staining (with Alcian Blue) of E14.5 embryos as well as bone and cartilage staining (with Alcian Blue and Alizarine Red) of E18.5 embryos were carried out as previously described [35]. For *lacZ* expression analysis E10.5 embryos were fixed for 15–30 min in 2% paraformaldehyde in PBS, while E14.5 embryos were fixed for 15–30 min in 4% PFA. X-gal staining was performed as described [35]. For detection of β-gal on limb sections, E10.5 and E11.5 FLs and HLs were fixed with 4% paraformadehyde for 8–12 hrs at 4°C, washed in PBS, cryoprotected with 30% sucrose, frozen at −70°C, sectioned (thickness 11 µm) and stained as described [35].

### Whole-mount RNA *in situ* Hybridization

For RNA:RNA whole-mount *in situ* hybridization (WMISH), embryos at the desired age were dissected in cold RNAse-free PBS, fixed in cold 4% PFA for 12–16 hrs, rinsed with PBS, permeabilized by treatment with proteinase-K, prehybridized and hybridized as previously published [60]. Digoxygenin (DIG)-UTP (Roche)-labeled antisense RNA probes were used, synthesized by in vitro transcription with conventional methods. WMISH was carried out following described procedures [64], the signal was detected using an alkaline phosphatase-conjugated anti-DIG antibody and developed with the chromogenic mix NBT-BCIP (Roche). With each probe, at least two normal and two mutant specimens were examined. The *Dlx5* probe comprised 780 bp and was linearized with EcoRI and transcribed with T7 RNA polymerase [57]. The *Dlx6* probe is a 350 bp fragment spanning exons 3–4 [53]. The *Msx1* probe was a 550 bp 3′ spanning the homeodomain, containing exon 1; the *Msx2* probe corresponded to 378 bp in the first exon of *Msx2* cDNA, the *Fgf8* probe corresponded to most of the mouse coding sequence, the *Bmp4* probe (a kind gift from B. Hogan) contains the 3′ UTR and most of the coding sequence from a mouse cDNA [65], the probe for *Gremlin* corresponded to the entire murine coding sequence (a kind gift from R. Zeller). After hybridization, the signal was revealed with the NBT-BCIP chromogenic reaction.

### RNA Quantification by Real-time PCR

Embryonic FLs and HLs at the age E11 were dissected in cold RNase-free PBS, the anterior and posterior halves were separated and pooled in RNA-later (Ambion). Pools of two half limbs from the same embryos were used to extract total RNA, using the Tissue Lyser II reagent followed by elution through RNA micro-kit plus (Qiagen). cDNA synthesis was done using standard conditions, 3 ng of each cDNA sample were used to carry out qPCRs on a CFX96 equipment (Biorad) using the SybrSafe system (Invitrogen). Samples were analyzed in technical triplicates, and for each genotypes (except for the *Msx1^+/−^;Dlx5^−/−^;Dlx6* ^−/−^) biological triplicates could be analyzed. Primer sequences were designed with the Primer Express online tool. RNA quality, primer efficiency and correct size were tested by RT-PCR and agarose gel electrophoresis. Standard curve were performed using WT cDNA with four calibration points: 1∶10; 1∶40; 1∶160; 1∶640. Specificity and absence of primer dimers was controlled by denaturation curves. *Rps9* mRNA abundance was used for normalization (primer sequences provided in Table S1).

### Genome-wide Identification of Dlx Binding Sites and Target Genes

We used the Position-Weight matrix (PWM) provided by JASPAR under accession PH0024.1. The score of a site was computed with standard log-likelihood ratios, using as null model the nucleotide frequencies computed over the whole intergenic fraction of the mouse genome. We considered for further analysis putative sites scoring 50% of the maximum possible score or better. We selected among the sites identified above the ones that are conserved in at least two of 8 vertebrate species (genome sequence version in parenthesis): mouse (mm9), human (hg19), cow (bosTau4), opossum (monDom5), platypus (ornAna1), chicken (galGal3), frog (xenTro2), zebrafish (danRer6) and lamprey (petMar1). A site is defined as conserved with species S if it lies in a region of the mouse genome which is aligned with a region of the S genome and the aligned sequence in/S/is a site according to the same definition used for mouse sites. All genomic sequences and pre-computed “Net” alignments were obtained from UCSC.

A ranked list of putative Dlx target genes was obtained from the sites determined above by associating each site conserved in at least one species to its closest Refseq mRNA, and then selecting the sites located either within 10 kb upstream of the TSS, or within the non-coding portion of the first exon, or in the first intron. We then associated to each putative target a score equal to the sum of the conservation scores (number of species) of its associated sites.

### Chromatin Immunoprecipitation

A *DLX5-myc* expression vectors (OriGene, USA) containing the full-length human *DLX5* cDNA with an in-frame insertion of the myc-TAG at the C-terminus, was used as described [66]. The Q178P point mutation [31] was inserted in the *DLX5-myc* expression vector indicated above, by site directed mutagenesis and sequence verified (Bio-Fab Research, Rome).

The U2Os human osteosarcoma cells were used; these cells express low or undetectable levels of *DLX5* mRNA endogenously, but have been shown to respond to Dlx expression with activation of the p63 promoter [57]. Eight µg of the *DLX5-*myc expression vectors were used for transfections, which yielded an efficiency of 35% (number of myc-positive cells over total counted nuclei). Chromatin was crosslinked, sonicated, immunoprecipitated with either the anti-myc TAG (A-14 sc-789, SantaCruz, USA) or the anti-acetyl-Histone H4 (06-866, ChIP Grade, Upstate Biotechnology USA,) antibodies and de-crosslinked according to instructions (EZ Magna ChipG, Millipore). Fragments of the human *BMP2* and *BMP4* loci spanning the identified conserved regions were PCR-amplified and analysed by gel electrophoresis (sequences provided in Table S2). Total chromatin was used as positive control (input), chromatin from cells transfected with an empty vector was used as negative control.

## Results

### Msx and Dlx Coexpression Analyses, *in silico*

Previous evidences indicate that Dlx5 binds to homeodomain-responsive elements in a proximal region of the *Msx2* promoter, and thereby regulates its transcription [62], [63]. To further support this possibility, we have used a human-mouse co-expression network, generated using published profiling datasets [67], [68] and found that *DLX5* and *MSX* genes are connected, i.e. each ranks in the first 1% of the co-expression lists of the other (p<0.01, data not shown).

Next, we screened conserved regions of the vertebrate genome for the presence of consensus Dlx DNA-binding sites, as defined by the Dlx5 PWM [69] present in the Jaspar database (accession N° PH0024.1) [70] and reported in Table S3. As the PWM for Dlx5 reported in Jaspar is not highly informative, it is not surprising that a total 565,995 putative binding sites were initially identified in the mouse genome. However, by introducing evolutionary conservation with at least two (out of 8) species examined as a further criteria, this number is reduced to 11,262 sites, and with conservation in three species is further reduced to 4085 (Table S3, complete lists available upon request). The full annotation on the UCSC genome browser is available upon request. As positive controls, the well defined Dlx sites present in the *Dlx5;Dlx6* intergenic region [71] and in the *Msx2* promoter [62], [63] were correctly predicted (Fig. S1). The evolutionary conservation of the sites suggests the presence of positive selection pressure to maintain the sites, confirming their functional relevance.

We then generated a ranked list of putative Dlx targets, based on the position of predicted conserved Dlx sites in the genome, as indicated in the Methods sections. The ranked list contains 3,051 Refseq mRNAs associated to 2,412 unique Entrez gene IDs (available upon request). The *Msx1* and *Msx2* genes were found in the top 10% of the list of putative Dlx targets, strengthening the possibility that Dlx proteins might directly regulate *Msx* expression.

### Expression of Msx and Dlx Genes during Limb Development

**Figure 1:**
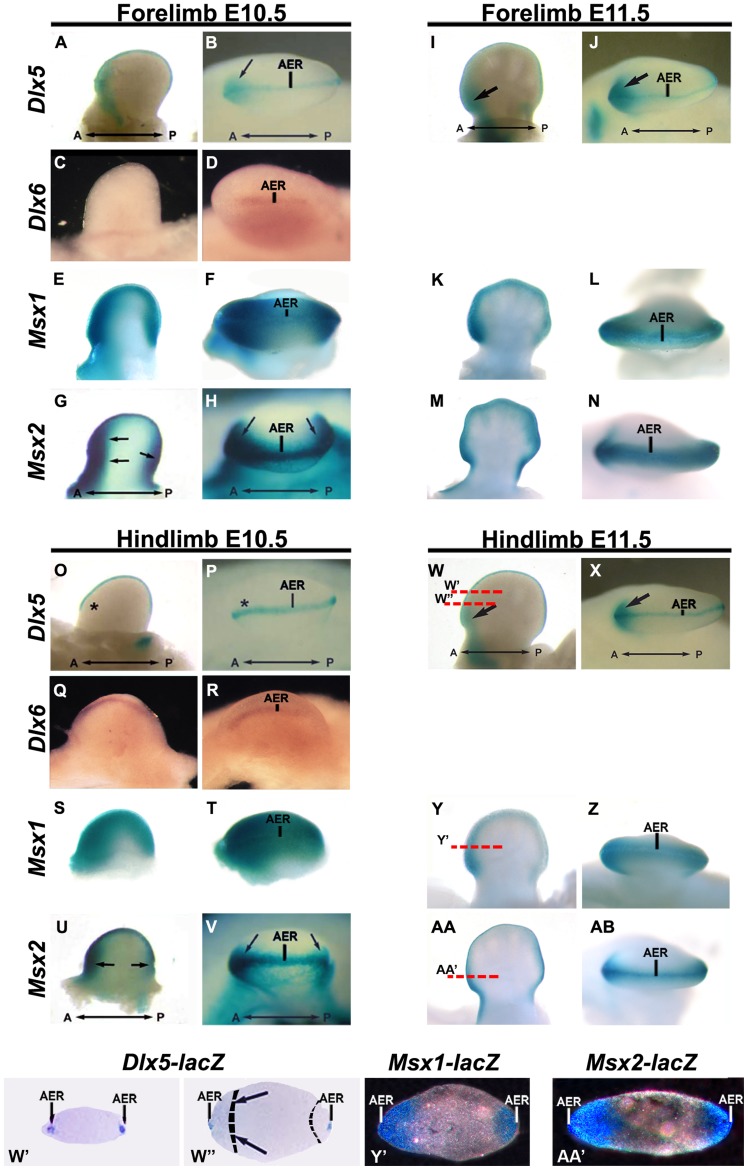
Spatio-temporal expression of *Dlx*-*Msx* in developing limbs. **A-N**, forelimbs. **O-AB**, hindlimbs, at E10.5 (A-H, O-V) and E11.5 (I-N, W-AB). Whole-mount X-gal staining on FLs and HLs from *Dlx5^+/−^, Msx1^+/−^* and *Msx2^+/−^* (*lacZ*+) heterozygous embryos are shown. Expression of *Dlx6* was detected by WMISH on embryonic limbs at the same ages and shown. At E10.5 *Msx2* and *Msx1* are expressed in the AER and the anterior and posterior mesoderm of HLs and FLs. *Dlx5* and *Dlx6,* at E10.5, are expressed in the AER of HLs and FLs and in the anterior limb mesoderm only of the FLs, but not of the HLs. At later stages (E11.5), *Dlx5* and *Dlx6* are then expressed in the anterior mesoderm of HLs. Black arrows indicate mesodermal expression. The AER is also indicated. Black asterisks indicate absence of expression. **W’,W’’** histologic transversal sections of E11.5 HLs from *Dlx5^+/−^* embryos, stained with Xgal. **Y’**,**AA’** histologic transversal sections of HLs from *Msx1^+/−^* (Y’) and *Msx2^+/−^* (AA’) embryos, to compare AER and mesodermal expression between these genes. Section planes and position are reported with red lines (in W, Y and AA). The extent of the *Msx1*-positive anterior and posterior mesoderm regions, based on the micrographs in AA’ and AC’, are indicated with dashed lines. A strong *Dlx5*-lacZ signal is detected in the AER (W’ and W’’), a weak *Dlx5*-lacZ signal, overlapping with the *Msx1*-lacZ and the *Msx2*-lacZ signal, is detected in the anterior mesoderm (W’’, indicated by black arrows).

We examined expression of *Dlx5, Dlx6, Msx1* and *Msx2* in the FL and HL of normal embryos, at E10.5 and E11.5, by X-gal staining of heterozygous embryos carrying an allele with inserted *lacZ* reporter (*Dlx5, Msx1, Msx2*), and by WMISH (*Dlx6*) (Fig. 1). WMISH for *Dlx5, Msx1* and *Msx2* have been previously reported with comparable results. In the AER, all four genes are co-expressed starting at E9.5/E10. On the contrary, in the limb mesoderm they are expressed with a different time-of-onset. At E10.5 *Msx1* and *Msx2* are mainly expressed in two mesoderm territories (anterior and posterior) of both the FL and the HL (Fig. 1E–H, S–V), whereas at the same stage the *Dlx5* transcript is detected only in the anterior mesoderm of the FL, and not that of the HL (compare Fig. 1A–B with O–P). The *Dlx6* transcript is not detected in the mesoderm at this stage (Fig. 1C–D, Q–R). In the HLs, mesodermal expression of *Dlx5* starts around E11.5 and is confined to the anterior margin (Fig. 1W–X). At this stage, *Msx1* and *Msx2* are expressed in the AER and in a larger region underneath the AER (Fig. 1K–N, Y-AB). Histological sections of X-gal stained limb buds from *Dlx5* ^+/−^, *Msx1* ^+/−^ and *Msx2* ^+/−^ embryos (Fig. 1W’, W’’, Y’ and AA’) reveal that *Msx1* and *Dlx5* are truly co-expressed in the AER (strong signal in W’ and W’’) and in the anterior HL mesoderm (a weak signal is present in W’’, indicated with black arrows).

In summary, *Msx* expression precedes that of *Dlx* in the HL anterior mesoderm, consequently in this location *Dlx* genes are unlikely to regulate *Msx* gene transcription cell-autonomously and Dlx and Msx proteins are unlikely to interact in the anterior limb mesoderm, at early stages.

### *Msx2* Expression is Downregulated in *Dlx5;Dlx6* DKO Limbs

**Figure 2:**
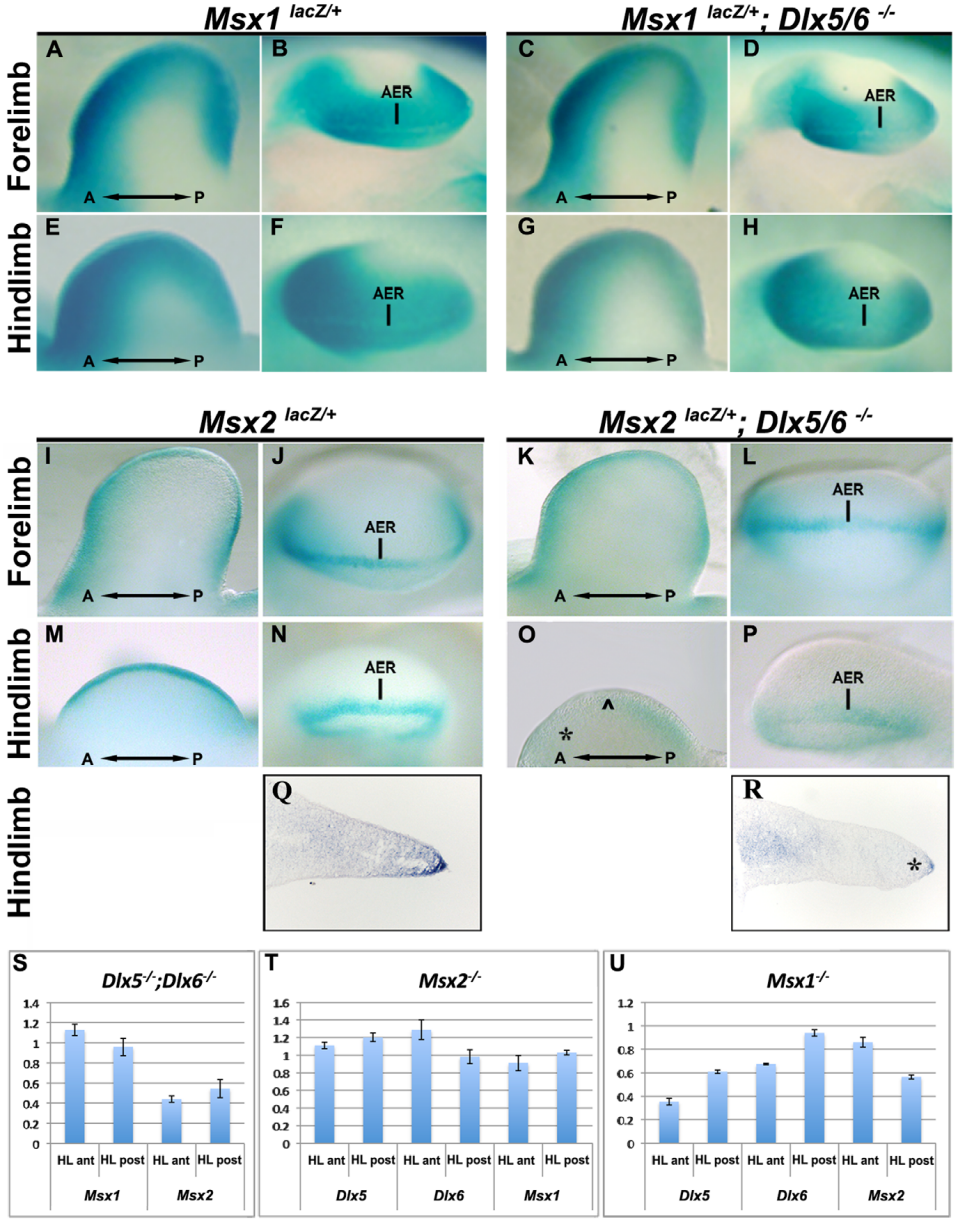
Reduction of *Msx2* expression in *Dlx5/Dlx6* DKO HLs. **A-P.** Whole-mount X-gal staining to detect *Msx1* and *Msx2* expression in *Dlx5;Dlx6* DKO. In the FL (A-D,I-L) no changes of expression is observed whereas in the HL (E-H, M-P), *Msx2* expression is reduced in the AER and in the anterior limb mesoderm of the *Dlx5;Dlx6* DKO HLs. **Q,R.** Sections of WT (Q) and *Dlx5;Dlx6* DKO mutant HLs (R) hybridized in situ to detect *Msx2*, showing a drastically reduced *Msx2* signal in the AER and in the underlying mesoderm, but not in a proximal mesoderm territory. **S-U.** Quantification of the expression of *Dlx5, Dlx6, Msx1* and *Msx2* mRNAs by qRT-PCR in HLs from *Dlx5;Dlx6* DKO (S), *Msx2^−/−^* (T) and *Msx1^−/−^* (U), relative to WT. The results show a reduction of 45% of *Msx2* expression in the *Dlx5;Dlx6* DKO HLs compared to WT, but not of *Msx1*. *Dlx5* and *Dlx6* expression is downregulated in *Msx1* KO HLs but not in *Msx2* KO HLs. Expression of the knocked-out genes was also tested, as control, and always found to be reduced to undetectable levels (not shown).

To investigate interactions between the *Dlx* and the *Msx* genes during limb development, we used animals DKO for *Dlx5;Dlx6* and analyzed the expression of the *Msx-lacZ* reporter in this genetic background. To do this, we compared the expression patterns of the *lacZ* reporter between *Msx1^+/−^* and *Msx1^+/−^*;*Dlx5^−/−^;Dlx6^−/−^* mice (Fig. 2A–H), and between *Msx2^+/−^* and *Msx2^+/−^*;*Dlx5^−/−^;Dlx6^−/−^* mice (Fig. 2I–P). We observed a clear reduction of *Msx2* expression in the anterior mesoderm and AER exclusively in the HLs, whereas neither expression of *Msx1* in the HLs nor expression of *Msx1* and *Msx2* in the FLs were significantly changed (Fig. 2A–P). To further document the reduction of *Msx2* expression in the AER of *Dlx5;Dlx6* DKO limbs, we carried out WMISH for *Msx2* on cryostatic sections of *Dlx5;Dlx6* DKO HLs, at E11. *Msx2* expression was strongly reduced or absent in the AER and the underlying mesoderm of a central wedge of the mutant limbs, as compared to the same region of normal limbs (Fig. 2Q,R).

Since WMISH is not a quantitative method, we used quantitative Real Time PCR (qRT-PCR) to quantify the reduction of *Msx2* mRNA on samples extracted from the HLs of normal and *Dlx5;Dlx6* DKO embryos, at E11.5. Limb buds were divided in two halves on the proximo-distal axis, to determine gene expression in the anterior and posterior mesoderm separately. In such samples, the AER cells contributed minimally, while most of the RNA derives from the mesenchyme. *Msx2* mRNA abundance was reduced by 60% in the anterior half, and by 50% in the posterior half of *Dlx5;Dlx6* DKO HLs from the same embryo, as compared to WT. In the same samples, expression of *Msx1* was minimally or not changed (Fig. 2S). We then determined the abundance of *Msx* and *Dlx* mRNAs in the anterior half of the HLs of *Msx1^−/−^* and *Msx2^−/−^* (single homozygous) mutant embryos, as compared to WT. In the *Msx2^−/−^* mutant the expression level of *Dlx5*, *Dlx6* and *Msx1* was unchanged, whereas in the *Msx1^−/−^* mutant the expression levels of *Dlx5*, *Dlx6* and *Msx2* were reduced by 65%, 40% and 15%, respectively. Moreover, in the posterior half of the HLs from the *Msx1^−/−^* mutant we observed a reduction of 40% in the abundance of *Dlx5* and *Msx2* mRNA, whereas *Dlx6* did not change (Fig. 2T,U).

### Limb Phenotype of *Msx2;Dlx5;Dlx6* Triple Mutants

To reveal possible functional interactions between the Dlx and the Msx gene products during limb development, we generated TKO mice with genotype *Msx1^−/−^;Dlx5^−/−^;Dlx6^−/−^* or *Msx2^−/−^;Dlx5^−/−^;Dlx6^−/−^.* TKO newborns were obtained at a frequency lower than expected, and all died shortly after birth, due to severe craniofacial malformations and consequent breathing impairment. Quadruple knock-out embryos were never obtained in spite of several attempts.

**Figure 3:**
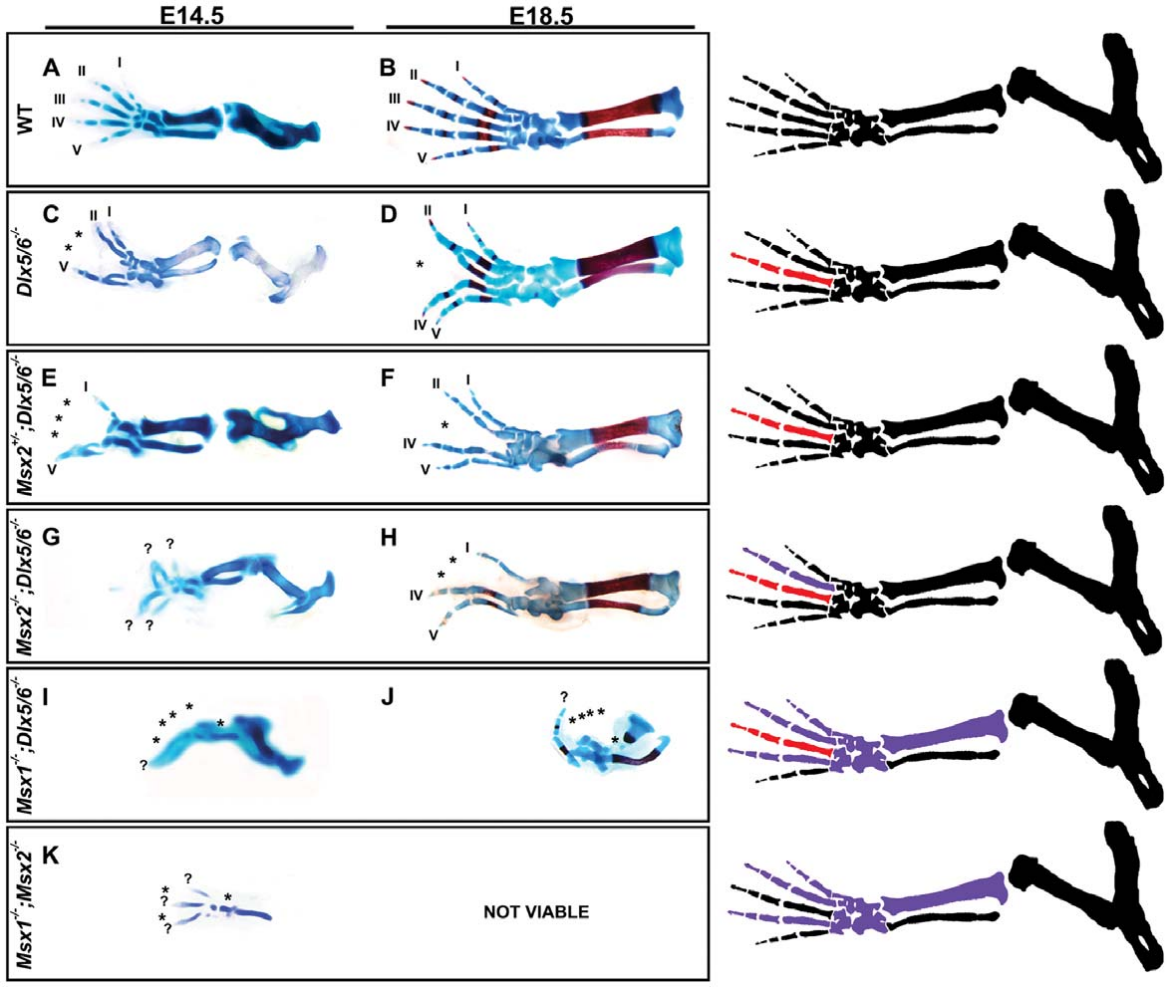
Skeletal preparations of the HLs of single and combined *Msx;Dlx* mutant animals. Chondroskeletal preparation of the HLs of E14.5 embryos (micrographs on the left) and full skeletal preparation on newborn animals (micrographs on the right), representing single and combined *Dlx*;*Msx* mutant genotypes (indicated on the left). The HLs of *Msx2* ^+/−^;*Dlx5;Dlx6* DKO animals (E,F) display an aggravated ectrodactyly phenotype compared to *Dlx5;Dlx6* DKO ones (C,D), with fusion of the external digit (1with 2, 4 with 5) and hypoplasia of the central digit. *Msx2*;*Dlx5*;*Dlx6* TKO HLs (G,H) display a further aggravated ectrodactyly phenotype, with the external digits fused and extended towards the opposite (anterior-posterior) sides, and a complete absence of the central digit. The limbs of *Msx1^+/−^;Dlx5;Dlx6* DKO mice (not shown) show ectrodactyly similar to that observed in *Dlx5;Dlx6* DKO mice, whereas *Msx1;Dlx5;Dlx6* TKO mice (I,J) show ectrodactyly and loss of skeletal elements deriving from the anterior mesoderm of the autopod and zeugopod, a phenotype seen in *Msx1;Msx2* DKO mutant embryos (K). The stylopod shows no evident defects. The anterior-posterior orientation is shown. The numbers 1–5 indicate the digits (1 is the toe). Asterisks indicate hypoplasia or absence of skeletal structures. The drawings on the left schematically illustrate the Dlx-related (red elements) and the Msx-related (purple elements) skeletal defects, corresponding to the genotypes examined.

We examined the limb skeleton in the TKO animals at two ages: E14.5 (with Alcian-Blue) and E18.5/birth (with Alcian-Blue and Alizarine-Red, which stain, respectively, cartilages and mineralized bones). In *Msx2;Dlx5;Dlx6* TKO animals, the HLs were severely affected (Fig. 3G,H) whereas no evident alteration was observed in the FLs (data not shown). The central digit was always missing, rarely (<10%) the two central digits were missing, while the remaining digits extended pairwise towards the opposite (anterior-posterior) sides. Importantly, no loss of anterior digits or of zeugopod elements was ever observed (Fig. 3G,H). The limb phenotype observed in the *Msx2;Dlx5;Dlx6* TKO mutant animals is consistent with a significant aggravation of the ectrodactyly phenotype seen in *Dlx5;Dlx6* DKO [29], [30] (compare Fig. 3G with 3C, and 3H with 3D), without appearance of other recognizable phenotypes. Remarkably a very similar phenotype, although less severe, was observed in animals with genotype *Msx2^+/−^;Dlx5^−/−^;Dlx6^−/−^*, i.e. in the presence of a single wild-type *Msx2* allele (Fig. 3E,F).

### Limb Phenotype of *Msx1;Dlx5;Dlx6* Triple Mutants

In *Msx1*;*Dlx5;Dlx6* TKO mice the FL were usually normal, although in one case (1/6) anterior polydactyly was observed (data not shown). Of note, polydactyly has been reported for the FLs in *Msx1;Msx2* DKO mice with a moderate penetrance [32]. On the contrary, the TKO HLs displayed a severe phenotype consisting in the loss of the tibia and of 3–4 preaxial digits (Fig. 3I,J). Since the HLs of *Msx1*;*Msx2* DKO usually exhibit loss of 1–2 anterior digits and of the tibia [32], we interpreted the TKO phenotype as a combination of ectrodactyly, caused by the loss of *Dlx5;Dlx6* [29], [30] (Fig 3C,D), and the preaxial adactyly as observed upon the loss of *Msx1;Msx2* [32] (Fig. 3K).

The appearance of features of the *Msx1;Msx2* DKO phenotype in the *Msx1;Dlx5;Dlx6* TKO animals, which retain two functional *Msx2* alleles, argues in favor of a severe reduction of *Msx2* expression in the TKO limbs, as compared to the *Dlx5;Dlx6* DKO.

Indeed, removing a single *Msx1* allele in the context of *Dlx5;Dlx6* DKO background (i.e.*Msx1^+/−^;Dlx5^−/−^;Dlx6^−/−^* embryos) results in a 50% reduction of *Msx2* mRNA in both the anterior and the posterior half of the embryonic HLs, as compared to WT. Moreover, expression of *Msx2* in the anterior and posterior halves of *Msx1* homozygus mutant HLs was reduced by 30% compared to *Msx1* heterozygous HLs (data not shown). This indicates that reduced *Msx2* expression may result from the combination of (1) loss of *Dlx5* and *Dlx6* (Fig. 2U, Fig. S2), upstream regulators of *Msx2*, and (2) the loss of *Msx1*, with the consequent decrease of *Msx2* expression (Fig. 2V).

### Expression of *Bmp4* and Gremlin in *Dlx5;Dlx6* Mutant Limbs

The phenotype of the *Msx1;Dlx5;Dlx6* TKO HLs shares some similarities with the *Msx1;Msx2* DKO phenotype [32]. However, defects in the anterior autopod and zeugopod cannot be simply explained by a direct *Dlx5;Dlx6* control on *Msx2* expression, since in the presumptive territory of the anterior zeugopod and autopod the expression of *Msx1* and *Msx2* precedes that of *Dlx5* and *Dlx6* (Fig. 1). Therefore we hypothesized that a non-cell autonomous regulation should take place, linking a defective AER with *Msx2* misexpression in the anterior limb mesoderm. We focused on Bmps as candidate signaling molecules since they are expressed in the AER as well as the anterior and posterior mesoderm [21], and since BMP signaling is known to induce *Msx* expression in several embryonic territories [20], [72].

**Figure 4:**
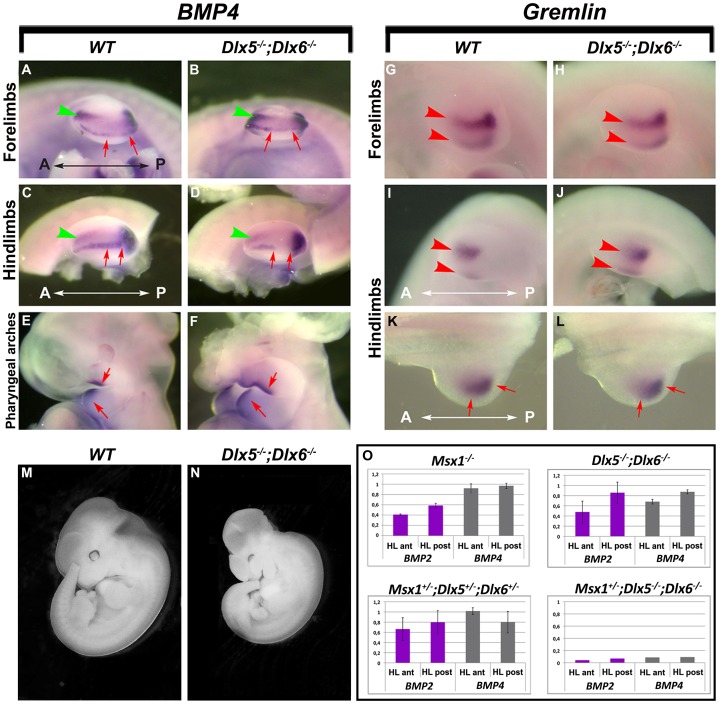
Expression of *Bmp4* in *Dlx5;Dlx6* DKO limbs. **A-D.** Detection of *Bmp4* mRNA by WMISH on WT (left) and *Dlx5;Dlx6* DKO mutant (right) limbs, at E11. FLs are on the top, HLs are on the bottom. **E,F.** *In situ* detection of *Bmp4* mRNA in the pharyngeal arches region of WT (left) and *Dlx5;Dlx6* DKO mutant (right), at E11, as a control for RNA preservation. **G-L.** Detection of *Gremlin* mRNA in FLs (G,H) and HLs (I-L) of WT (left) and *Dlx5;Dlx6* DKO mutant embryos (right), at E10.5. While *Bmp4* expression in the anterior mesoderm of the FLs (A,B) or the HLs (C,D) is unchanged (green arrowheads), expression in the central wedge of the AER of mutant embryos is diminished in the HLs, but not in the FLs (red arrows in D). *Gremlin* expression is unchanged both in the FLs (G,H) and in the HLs (I-L) of Dlx mutant embryos (red arrowheads). Genotypes and probes are reported on the top. The Anterior-Posterior (A-P) orientation is indicated. **M,N.** whole-mount photographs documenting the reduced size of mutant embryos and justifying the slightly reduced size of the mutant limbs, often observed. **O.** Quantification of *Bmp2* and *Bmp4* mRNAs by qRT-PCR in the anterior and posterior halves of HLs from embryos with the genotype indicated on the top of each graph, compared to WT.

We carried out WMISH for *Bmp4* on *Dlx5;Dlx6* DKO embryos at E11 and found that expression is decreased specifically in the central sector of the AER of the mutant HLs (3 of 4 embryos) but not significantly in the anterior mesoderm (Fig. 4C,D). *Bmp4* expression was not significantly changed in the FLs of the same embryos (Fig. 4A,B), as expected considering the lack of any morphological defects in the FLs. As a further control, *Bmp4* expression was retained in the pharyngeal arches region (Fig. 4E,F). We also examined the expression of *Gremlin* in the limbs of E10.5 embryos. Gremlin is a BMP antagonist that participates in a regulatory loop between *Shh* in the posterior limb mesoderm and *FGF4* in the posterior AER, which maintains the AER and ZPA and promotes digit patterning and limb outgrowth [17], [73], [74]. The anterior-posterior patterning and expression level of *Gremlin* did not significantly change in either the FLs or the HLs of *Dlx5;Dlx6* DKO embryos (Fig. 4G–L). Thus, we concluded that the inactivation of *Dlx5* and *Dlx6* does not lead to significant alterations of limb antero-posterior patterning. Finally, the expression of *Bmp7* was not significantly changed (data not shown).

### *Bmp2* and *Bmp4* Expression Levels are Synergistically Reduced by Msx and Dlx Mutations

The WMISH results reported above suggest that *Bmp4* expression is unchanged in the anterior mesoderm of the limbs; however this technique is poorly quantitative and cannot reveal minor changes in gene expression level. To further investigate the mechanism that might induce and/or sustain *Msx2* expression in the anterior half of the HLs, we determined the relative abundance of *Bmp2* and *Bmp4* mRNAs by qRT-PCR in the anterior and posterior halves of the embryonic HLs (E11) with different genotypes. Expression of *Bmp2* is mainly decreased in the anterior (about 50%), and minimally in the posterior half (20%) of the HLs from *Dlx5;Dlx6* DKO embryos (Fig. 4O). *Bmp2* expression is similarly reduced in *Msx1^−/−^* HLs. *Bmp4* expression is minimally or not affected in the *Msx1^−/−^* mutants, while in the *Dlx5*;*Dlx6* DKO *Bmp4* expression is reduced of about 30% and 20%, respectively, in the anterior and in the posterior halves (Fig. 4O). Of note, *Bmp4* expression is also reduced in *Msx2^−/−^* embryonic HLs, less severely in the anterior (30%) than the posterior half (50%) (data not shown). Strikingly we observed a strong synergy between *Msx1* and *Dlx5;Dlx6* in controlling BMP expression. When combined with the *Dlx5;Dlx6* DKO mutation, the loss of a single *Msx1* allele was sufficient to reduce *Bmp2* and *Bmp4* expression to levels lower than 10% of that of control samples, both in the anterior and in the posterior halves of the HLs. Considering that *Msx2* is a known target of Bmp2 and Bmp4 in several tissues [72], [75], [76], this may explain how *Msx2* expression is reduced in the *Msx1*;*Dlx5*;*Dlx6* TKO, and how this mutant shows a limb phenotype with similarities to the *Msx1*;*Msx2* DKO.

Finally, we determined the abundance of *Fgf8* and *Shh* mRNAs in HL samples from the relevant genotypes (Fig. S3). The Abundance of *Fgf8* mRNA was slightly reduced in the absence of *Dlx5* and *Dlx6* (DKO samples), and was further drastically reduced when one *Msx1* allele was eliminated in the absence of *Dlx5;Dlx6*. Similarly, the abundance *Shh* mRNA (in the posterior half of the limb buds) was slightly reduced in the absence of *Dlx5* and *Dlx6*, and was further reduced when one *Msx1* allele was simultaneously eliminated. While reduced *Fgf8* expression is not surprising [30], reduced *Shh* expression is novel and may contribute to the altered digit number and morphogenesis seen in the *Msx1,Dlx5;Dlx6* TKO embryos.

### Dlx5 Binds to Conserved Sequences in the Proximity of the *Bmp2* and *Bmp4* Loci

**Figure 5:**
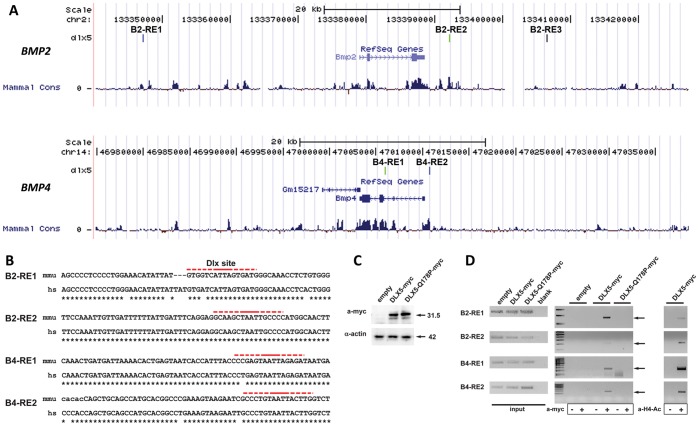
Binding of DLX5 on predicted conserved elements close to *BMP2* and *BMP4*. A. Location of predicted conserved Dlx binding sites in regions of the mammalian genome around the *BMP2* (top) and *BMP4* (bottom) loci. Sites are indicated with colour vertical bars, the chromosomal position and coordinates are shown. The mammalian genomic conservation is reported on the bottom. With the exception of B2-RE3, all elements fall within stretches of conserved sequences. **B.** Sequences and alignment of the predicted Dlx binding elements. The sequence corresponding to the PWM is shown in red. Dashed lines indicate the degenerated part of the binding sequence. **C.** Western blot analysis to demonstrate expression of DLX5-myc and DLX5-Q184P-myc proteins in U2Os cells. The molecular weight of the detected proteins is indicated on the left. **D.** ChIP analyses on the predicted Dlx binding sites in the human U2Os cells, transfected with the *DLX5-myc* and *DLX5-Q178P-myc* expression vector, or with the control empty vector. The input chromatin (positive control) is shown on the left, ChIP with or without anti-myc are shown in the mid panels. ChIP with and without anti-H4Ac on the same elements are shown on the right.

To search for possible direct regulations of the *Bmp2* and *Bmp4* loci by Dlx5, we first exploited the genome-wide predictions of conserved Dlx sites, described above. In the proximity of the *Bmp2* locus we identified three Dlx consensus binding sequences, named B2-RE1, located 30 kb upstream of the transcription start site (TSS), B2-RE2 and B2-RE3, located respectively 3.5 kb and 18 kb downstream of the end of the *Bmp2* transcript. All these Dlx binding elements are conserved, although not identical, in at least two vertebrate species, and fall within stretches of conserved genomic sequence (Fig. 5A,B). The B2–R3 element was recognized as conserved only between human and mouse, and contains 6 (of 16 bases) mismatches, in non-critical positions. In the proximity of the *Bmp4* locus we identified two Dlx consensus sequences, named B4-RE1 and B4-RE2, located respectively 430 bp upstream of the TSS and within the second intron, conserved in at least two mammalian species (Fig. 5A,B). Also these predicted Dlx binding sites fall within stretches of conserved genomic sequence (sequences and genomic locations provided in Tables S4 and S5).

To demonstrate that the Dlx5 protein physically binds to these putative responsive elements, we transfected a human *DLX5*-myc-tag expression vector [66] and the same one modified to harbor the disease-causing point mutation Q178P [31] in the U2Os osteoblast cells (Fig. 5C). These cells were chosen since they are of human origin, they express low endogenous level of *DLX5*, have been shown to respond to *Dlx5* expression by activation of the *p63* and other promoters [57], are of osteoblastic origin (*Dlx5* plays a role in late osteoblast differentiation [35]), and are easily transfected. The crosslinked chromatin was immunoprecipitated with an anti-myc antibody, or with anti-acetylated Histone 4 (H4Ac) and subjected to PCR amplification with primers flanking the predicted DLX sites in the human genome. With the exception of the B2-RE3 element (not shown), all the other elements showed an enrichment of PCR amplification products from chromatin of DLX5-myc transfected cells, as compared to the chromatin from cells transfected with the empty vector (Fig. 5D). The same elements immunoprecipitated with anti-H4Ac, suggesting that in these locations the chromatin is available for transcription regulation. Interestingly, the Q178P mutant DLX5 protein did not show any binding to these sequences, thus this disease-causing mutation is associated with loss of DNA-binding activity on the Bmp elements. As further controls, PCR amplification of two sequences containing no identifiable homeodomain-binding sequences (*BMP2* exon 3 and *BMP4* exon 5), as well as an irrelevant sequence (*IL10*) did not show any enrichment (not shown). These results reinforce the notion that DLX proteins physically interact with four (out of five) conserved elements close to the *BMP2* and the *BMP4* loci, and might exert a direct transcription regulatory activity, relevant for normal limb development.

## Discussion

*Dlx5*, *Dlx6, Msx1* and *Msx2* genes encode homeodomain transcription factors involved in limb development. The targeted disruption of each of these genes, individually, does not lead to limb defects, however double *Msx1/Msx2* [33], [34] and *Dlx5/Dlx6* [29], [30] mutant mice present severe limb malformations indicating that these genes participate in the control of digit number and morphogenesis. The Dlx and Msx homeodomains show a high degree of sequence similarity and similar DNA binding sequences *in vitro* [43]. In spite of these similarities, there is no evidence suggesting that these homeoproteins cooperate or interact, except in two specific developmental processes: elevation and fusion of the palatal shelves [60], [61] and development of the frontal bone [59]. In this work, we shed light on direct and indirect interactions between these two classes of homeodomain genes during limb development.

### Direct and Indirect Dlx-Msx Regulations during Limb Bud Development

Although *Msx2;Dlx5;Dlx6* TKO mice show an aggravation of the ectrodactyly phenotype, as compared to *Dlx5;Dlx6* DKO, they do not present any obvious additional defect. Furthermore, inactivation of either *Dlx5*;*Dlx6* or *Msx2* does not modify the level of *Msx1* expression, suggesting that the *Msx2;Dlx5;Dlx6* TKO phenotype represents the exclusive contribution of the absence of *Msx2* in the *Dlx5;Dlx6* mutant context. This leads us to conclude that either *Msx2* has a rather minor function in limb development, or that its function is largely compensated by *Msx1*, in agreement with the limited defects observed in *Msx1^+/−^;Msx2^−/−^* compound mutant animals [32] (Y. Lallemand, unpublished). On the contrary, *Msx1;Dlx5;Dlx6* TKO mice display a limb defect which can be interpreted as the sum of the limb anomalies found in the *Msx1;Msx2* DKO and in the *Dlx5*;*Dlx6* DKO. These results strongly suggest that the expression of *Msx2* is suppressed in this genetic context, and that therefore *Dlx5;Dlx6* are genetically upstream of *Msx2*.

We show that in *Dlx5;Dlx6* DKO limbs *Msx2* expression is diminished in the central sector of the AER. As starting at E10.5 *Dlx* and *Msx* genes are co-expressed in the AER, it is possible to hypothesize that in this territory Dlx proteins bind directly on the *Msx2* promoter, as previously reported [62], [63]. On the contrary, in the anterior limb mesenchyme, the expression of *Msx1* and *Msx2* precedes that of *Dlx5* and *Dlx6*. This precludes the possibility of a direct regulation of *Msx* genes by Dlx proteins. Nevertheless, our qRT-PCR analyses show that *Msx2* expression is reduced in the anterior limb mesoderm of *Dlx5;Dlx6* DKO limbs, implying the existence of a non cell-autonomous mode of regulation between the AER and the anterior limb mesoderm. It is possible, therefore, that a diffusible protein, expressed by AER cells in a Dlx-dependent fashion, is required to initiate and/or sustain *Msx2* expression in the anterior limb mesoderm.

### Bmps as Signaling Relays between Dlx and Msx

*Msx* genes are well documented downstream effectors of BMP signaling in several developing structures [72], [77], [78], [79], [80], [81], [82]. Enhanced BMP signaling in the limb in mice deficient for the BMP antagonist *Gremlin*, results in upregulation of both *Msx1* and *Msx2* expression [73]. Conversely, blocking BMP signaling in the limb ectoderm by ectopic expression of Noggin, another BMP antagonist, results in decreased *Msx2* expression [23]. In the chick limb buds, expression of a constitutively-active BMP receptor, or misexpression of *Msx1*, in the dorsal ectoderm, induces the formation of ectopic AERs [20]. In addition, the combined inactivation of *Msx1* and *Msx2* leads to a phenotype that mimics, in some aspects, the loss of BMP signaling [32].

Noticeably, *Msx* genes are also upstream of Bmp4 in several developmental systems. During tooth germ development, mesodermal Msx1 is needed for efficient *Bmp4* expression [83], [84], [85], [86]. Palatal development, which is impaired in *Msx1* ^−/−^ mice, can be rescued by a *Bmp4*-expressing transgene [87]. In the limb itself, *Msx* genes are required in the mesoderm for maintenance of *Bmp4* expression [34]. Thus, BMP signaling is both upstream and downstream of *Msx* genes, depending on the context and the developing structure. The possibility that Bmp2 and Bmp4 participate in a Dlx-Msx signaling loop between the limb bud AER and mesoderm is clearly in line with previously identified roles of these molecules.

Therefore, Bmps represented likely candidates to mediate a non cell-autonomous regulation between AER-expressed *Dlx5;Dlx6* and mesodermal *Msx2*. Indeed, we find that *Bmp2* and *Bmp4* expression is significantly reduced in *Dlx5;Dlx6* DKO hindlimbs, at E11 when no evident defect is yet visible. Reduction for *Bmp2* and *Bmp4* in the anterior part of the HL is too high (50 and 30%, respectively) to be accounted for by the ectoderm alone, and rather indicates a downregulation of these Bmps in the mesoderm, too. This could mean that Bmps produced in the ectoderm activate Bmps also in the mesoderm, either directly or via Msx1. Indeed, further inactivation of one *Msx1* allele in the context of the loss of *Dlx5;Dlx6* nearly abolishes *Bmp2* and *Bmp4* expression, necessarily implying both the ectoderm and the mesoderm (Fig. 4K). This downregulation would explain a reduction in *Msx2* expression in the anterior mesenchyme in the *Dlx5;Dlx6* mutant limbs, at the same embryonic ages. The direct action of Dlx over Bmp is further supported by our ChIP data which indicate that DLX5, but not its mutated variant Q178P [31], binds to conserved regions near the *BMP2* and *BMP4* loci. However formal evidence that DLX5 activates *BMP2* and *BMP4* transcription is still lacking, as no suitable AER-related cell line is available for such experiments. It would be of interest to further investigate whether MSX1 also binds to the same conserved sequences in the *BMP2* and *BMP4* promoters.

Such a non cell-autonomous mode of regulation is strikingly similar to that occurring during development of the palatal shelves and the tooth primordia, both of which involve diffusion of Bmp between adjacent epithelial/mesodermal cell layers [60], [87], and in the case of the tooth germ, an induction of *Bmp4* expression in the mesoderm by ectodermal Bmp4 via expression of the Bmp target Msx1 [85], [86].

The loss of mesenchymal *Bmp2, 4* and *7* expression, on the other side, has been shown to be required for osteogenic differentiation, in a dose-dependent fashion and with Bmp molecules acting in a partially redundant way [88]. In their work, the selective loss of *Bmp2* and *Bmp4* in the limb mesenchyme affects zeugopod development and skeletogenesis, and less severely the autopod. On a similar note, another work [22] shows that the gradual elimination of *Bmp4* from the limb mesenchyme is required to rescue the *Grem1^−/−^* phenotype and a normal digit organization, implying a right amount of mesenchyme-derived Bmps is essential for AER function and for autopod morphogenesis. In the *Msx1*;*Dlx5;Dlx6* TKO mutants we observe a quite severe autopod defect (loss of digits) accompanied, however, by less severe zeugopod defect. Thus, it appears that both AER- and mesenchyme-derived Bmps are involved in the *Msx;Dlx* defects. The possibility that misexpression of AER-Bmps alone directly cause the TKO defects is unlikely. Rather, in light of the well known self-regulatory system of signalling loops comprising FGFs, SHH and BMPs [22], and considering that we observe changes in the expression levels of *Fgf8* and *Shh* upon loss of *Msx1*, *Dlx5* and *Dlx6* genes, most likely the overall nature of the limb defects in TKO mutant embryos is a quantitative misregulation of the “slow module” of this loop. *Dlx* and *Msx* genes can be regarded as new players in this complex regulation. Since Shh participates in the “slow module”, a more critical role for it could be envisioned, as suggested by resemblance of the phenotype of *Msx1;Dlx5;Dlx6* TKO limbs with that exhibited by *Shh* KO embryos [89].

**Figure 6:**
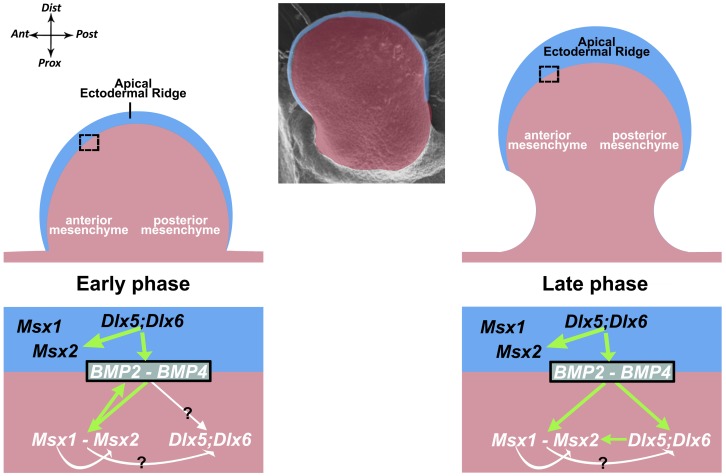
A dynamic model for Dlx-Msx-Bmp functional interactions during HL development. Schematic drawing to summarize our results and illustrate our model of functional interaction between *Dlx5;Dlx6*, *Msx1, Msx2, Bmp2* and *Bmp4*. On the top, a scheme of the limb bud, the AER (in light blue color) and the mesoderm (in pink color) is reported. Below, the proposed dynamic model of gene regulations, shown for an Early (E9.5–E10, on the left) and a Late (E10–E10.5, on the right) phases of HL development, using the same color code as above. The anterior mesenchyme is framed with a dotted black box; the Ant-Post and Prox-Dist directions are shown. Bmp2 and Bmp4 are placed at the interface between the AER and the Ant Mes, to indicate that these are diffusible signaling molecules.

In conclusion, we propose a model that involves a complex epithelial-mesodermal dialogue between Dlx and Msx (Fig. 6), entailing two distinct modes of regulation in the limb buds: a direct, cell-autonomous, regulation intrinsic to AER cells, and an indirect regulation between the AER cells and the anterior limb mesoderm. We further provide data suggesting that Bmp2 and Bmp4 mediate a non cell-autonomous control of Dlx over Msx, establishing a dialogue between the AER and the anterior mesoderm of the developing limb.

### Genotypes, Morphotypes, Gene Dosage and Expression Levels

When comparing the limb phenotypes of the genetic series *Dlx5;Dlx6* DKO vs. *Msx2^+/−^;Dlx5^−/−^;Dlx6^−/−^* vs. *Msx2;Dlx5;Dlx6* TKO (Fig. 3C–H), we observe a clear increase in severity of the ectrodactyly defect. This interesting observation can be explained by introducing the notion that *Msx2* expression depends on allelic dosage, and that a threshold level of *Msx2* expression is critical to drive normal morphogenesis.

The importance of allelic dosage, hence quantitative gene expression, is increasingly being recognized, even when phenotypes are not evident (see [88], [90]). As a further example, we detect a reduction of *Msx2* mRNA level in the *Msx2^+/−^* mice, in which no evident phenotype can be seen. Our explanation, in this case, is that reduced *Msx2* expression alone is not sufficient to cause limb malformations due to the presence of two functional *Msx1* alleles. Loss of one *Msx2* allele in the context of *Dlx5;Dlx6* DKO, instead, aggravates the phenotype. We explain this by proposing that *Msx2* expression is severely reduced, approaching that of the null condition, due to the combination of a) the genetic inactivation of one allele, and b) the lack of *Dlx5;Dlx6* genes.

Likewise, in *Msx1;Dlx5;Dlx6* TKO animals, *Msx2* expression is further reduced. A residual level is nonetheless observed; however, this is not sufficient to compensate for the loss of *Msx1*. In conclusion, allelic dosage and quantitative gene expression are crucial factors to be considered in the interpretation of a series of phenotypes, especially when related genes are involved.

### Conclusions

In human, the *DLX5* and *DLX6* genes cause the SHFM-type-1 congenital malformation when lost or mutated, while the *MSX1* and *MSX2* genes cause cleft palate and tooth agenesis. By crossbreeding mutant mouse strains, we show that the *Dlx5;Dlx6* and *Msx1;Msx2* genes cooperate for normal limb development and morphogenesis. At least two modes of regulation have emerged, one in which *Dlx5;Dlx6* control expression of *Msx2* cell-autonomously, the other in which the AER and the anterior mesenchyme interact non cell-autonomously, entailing Bmps as signaling molecules. We further show that the *BMP2* and *BMP4* loci comprise Dlx5-binding elements, occupied by Dlx5. Thus, the highly related homeodomain genes *Dlx* and *Msx* are two key players of a novel set of molecular and histological interactions during limb development.

## Supporting Information

Figure S1 **Top.** Location of predicted conserved Dlx binding sites in the *Dlx5-Dlx6* intergenic genomic region. Sites are indicated with colour vertical bars (asterisk) and annotated with the species conservation. The chromosomal position and coordinates are also reported. The mammalian genomic conservation is reported on the bottom. The known i56i element is correctly predicted by the PWM bioinformatic approach we have adopted. **Bottom.** Same as above, relative to the *Msx2* proximal promoter. Two known conserved Dlx binding sites are correctly predicted.
(PDF)

Figure S2 **Quantification of the** ***Msx2*** **mRNAs by qRT-PCR in the anterior and posterior halves of HLs from** ***Msx1+/−;Dlx5−/−;Dlx6−/−*** **embryos, relative to the corresponding WT samples (set = 1).**
(PDF)

Figure S3 **Quantification of the** ***Fgf8*** **and** ***Shh*** **mRNAs by qRT-PCR in the HLs from** ***Msx1−/− (top left), Dlx5−/−;Dlx6−/−*** **(top right),** ***Msx1+/−;Dlx5+/−;Dlx6+/−*** **(bottom left) and** ***Msx1+/−;Dlx5−/−;Dlx6−/−*** **(bottom right) embryos, relative to the corresponding WT samples (set = 1).**
(PDF)

Table S1 **Sequences of the oligonucleotides used for real-time qPCR on mouse embryonic tissues.**
(PDF)

Table S2 **Sequences of the oligonucleotides used for ChIP analysis on the predicted** ***Dlx*** **elements near the human** ***BMP2*** **and** ***BMP4*** **loci.**
(PDF)

Table S3 **The Dlx5 Position-Weight matrix and results of the prediction of Dlx5 binding sites based on genomic conservation.**
(PDF)

Table S4 **Sequences of the mouse and human conserved genomic regions containing predicted Dlx5 binding sites near the** ***BMP2*** **locus.**
(PDF)

Table S5 **Sequences of the mouse and human conserved genomic regions containing predicted Dlx5 binding sites near the** ***BMP4*** **locus.**
(PDF)
