# Supplementary figures and images for: BMP-Mediated Functional Cooperation between Dlx5;Dlx6 and Msx1;Msx2 during Mammalian Limb Development
Source: PLoS One. 2013 Jan 29;8(1):e51700. doi: 10.1371/journal.pone.0051700 (PMC3558506; doi:10.1371/journal.pone.0051700)

Suppl. Fig. S1

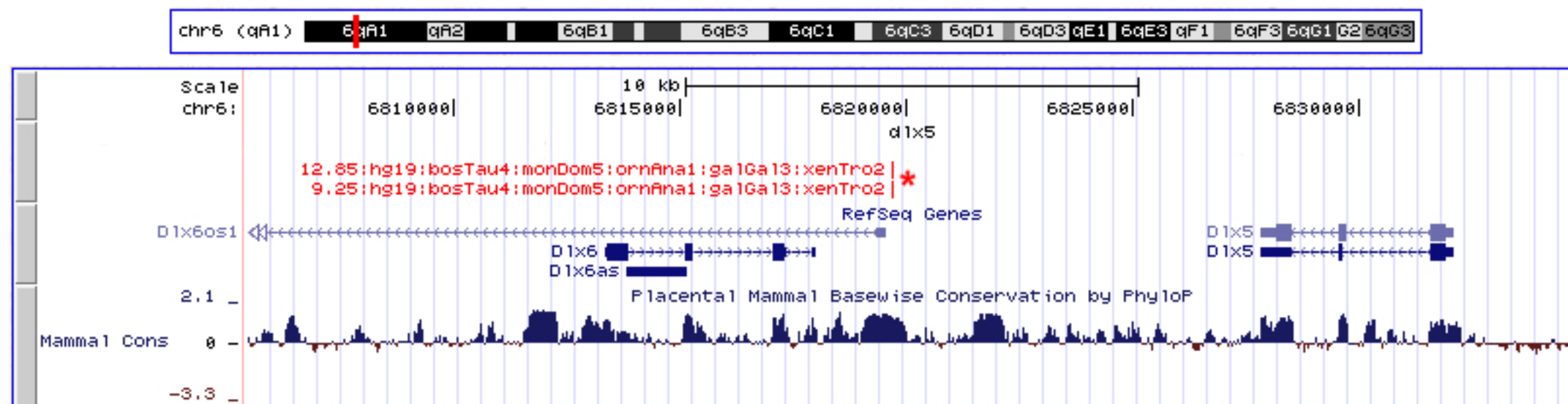

\* Regulatory elements contained in i56i enhancer region

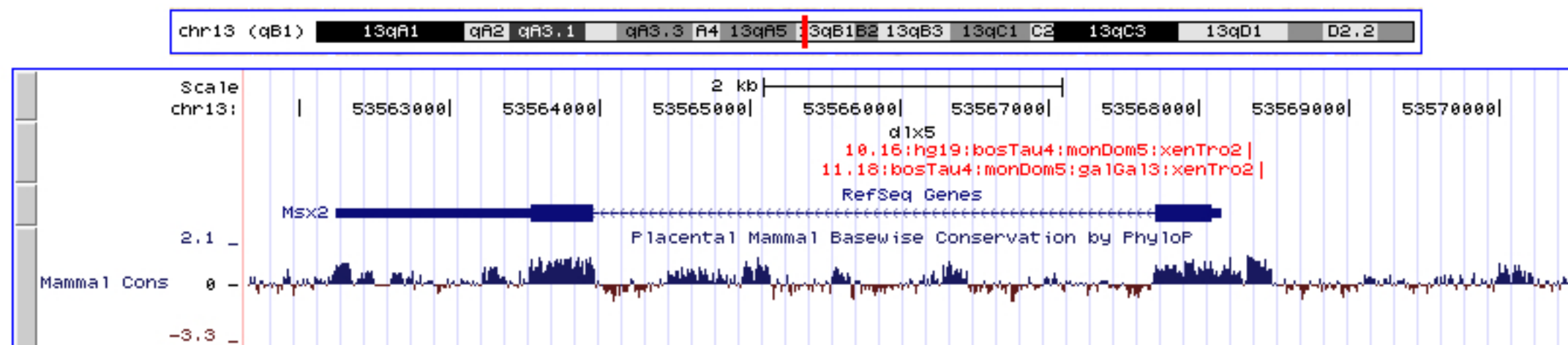

Supplement: Figure S1 — Top. Location of predicted conserved Dlx binding sites in the Dlx5-Dlx6 intergenic genomic region. Sites are indicated with colour vertical bars (asterisk) and annotated with the species conservation. The chromosomal position and coordinates are also reported. The mammalian genomic conservation is reported on the bottom. The known i56i element is correctly predicted by the PWM bioinformatic approach we have adopted. Bottom. Same as above, relative to the Msx2 proximal promoter. Two known conserved Dlx binding sites are correctly predicted. (PDF) [file pone.0051700.s001.pdf]

# Suppl. Fig. S2

***Msx1*<sup>+/-</sup>;*Dlx5*<sup>-/-</sup>;*Dlx6*<sup>-/-</sup>**

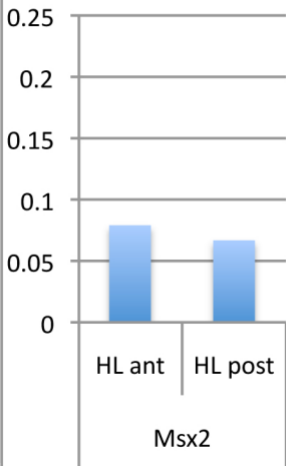

Supplement: Figure S2 — Quantification of the Msx2 mRNAs by qRT-PCR in the anterior and posterior halves of HLs from Msx1+/−;Dlx5−/−;Dlx6−/− embryos, relative to the corresponding WT samples (set = 1). (PDF) [file pone.0051700.s002.pdf]
