# Supplementary material for: BMP-Mediated Functional Cooperation between Dlx5;Dlx6 and Msx1;Msx2 during Mammalian Limb Development
Source: PLoS One. 2013 Jan 29;8(1):e51700. doi: 10.1371/journal.pone.0051700 (PMC3558506; doi:10.1371/journal.pone.0051700)
Supplement: Figure S3 — Quantification of the Fgf8 and Shh mRNAs by qRT-PCR in the HLs from Msx1−/− (top left), Dlx5−/−;Dlx6−/− (top right), Msx1+/−;Dlx5+/−;Dlx6+/− (bottom left) and Msx1+/−;Dlx5−/−;Dlx6−/− (bottom right) embryos, relative to the corresponding WT samples (set = 1). (PDF) [file pone.0051700.s003.pdf]

Suppl. Fig. S3

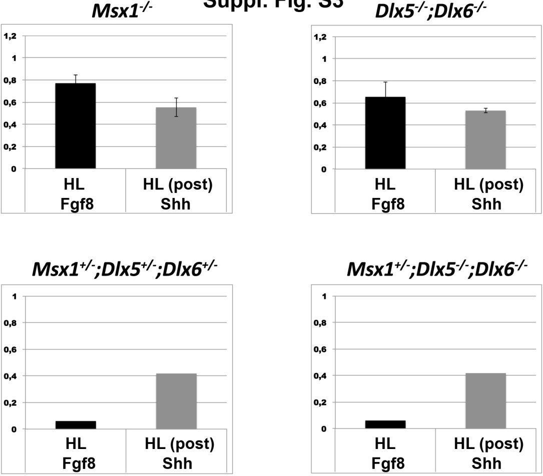

Msx1$^{-/-}$
1,2
1
0,8
0,6
0,4
0,2
0
HL
Fgf8
HL (post)
Shh
Dlx5$^{-/-}$*;Dlx6*$^{-/-}$
1,2
1
0,8
0,6
0,4
0,2
0
HL
Fgf8
HL (post)
Shh
Msx1$^{+/-}$*;Dlx5*$^{+/-}$*;Dlx6*$^{+/-}$
1
0,8
0,6
0,4
0,2
0
HL
Fgf8
HL (post)
Shh
Msx1$^{+/-}$*;Dlx5*$^{-/-}$*;Dlx6*$^{-/-}$
1
0,8
0,6
0,4
0,2
0
HL
Fgf8
HL (post)
Shh
