# Supplementary material for: BMP-Mediated Functional Cooperation between Dlx5;Dlx6 and Msx1;Msx2 during Mammalian Limb Development
Source: PLoS One. 2013 Jan 29;8(1):e51700. doi: 10.1371/journal.pone.0051700 (PMC3558506; doi:10.1371/journal.pone.0051700)
Supplement: Table S1 — Sequences of the oligonucleotides used for real-time qPCR on mouse embryonic tissues. (PDF) [file pone.0051700.s004.pdf]

Table S1

Sequences of the oligonucleotides used for real-time qPCR on mouse embryonic tissues

Rps9	For	5’ GACCAGGAGCTAAAGTTGATTGGA
Rps9	Rev	5’ TCTTGGCCAGGGTAAACTTGA
Dlx5	For	5’ TCTTATGGCAAAGCGCTCAA
Dlx5	Rev	5’ CGTTCACGCCGTGGTACTG
Dlx6	For	5’ TCCAGTGTGGGACGTTTCTG
Dlx6	Rev	5’ CTGTTGGGAGGCATACTGACG
Msx1	For	5’ CTACACCGCCCATGTAGGCTA
Msx1	Rev	5’ CTGGACCCACCTAAGTCAGGT
Msx2	For	5’ ATCCCAGCTTCTAGCCTTGGA
Msx2	Rev	5’ GACAGGTACTGTTTCTGGCGG
BMP2	For	5’ CAAAATCCCTAAGGCATGCTG
BMP2	Rev	5’ ATGGAGATTGCGCTGAGCTC
BMP4	For	5’ CGAGCCAACACTGTGAGGAGT
BMP4	Rev	5’ AGGTTGAAGAGGAAACGAAAAGC
