# Supplementary material for: BMP-Mediated Functional Cooperation between Dlx5;Dlx6 and Msx1;Msx2 during Mammalian Limb Development
Source: PLoS One. 2013 Jan 29;8(1):e51700. doi: 10.1371/journal.pone.0051700 (PMC3558506; doi:10.1371/journal.pone.0051700)
Supplement: Table S2 — Sequences of the oligonucleotides used for ChIP analysis on the predicted Dlx elements near the human BMP2 and BMP4 loci. (PDF) [file pone.0051700.s005.pdf]

Table S2

Sequences of the oligonucleotides used for ChIP analysis

on the predicted *Dlx* elements near the human BMP2 and BMP4 loci

**B2-RE1**
For: 5’ GACTCTCAAGGGAAATCTGAC – 3’
Rev: 5’ GGCACAGACTCAAACCTGG – 3’
Amplicon : 172 bp
**B2-RE2**
For: 5’ CCGCTTGGCCACAACACTATG – 3’
Rev: 5’ GGCCCAGCACTGTCAGGAAG – 3’
Amplicon: 224 bp
**B2-RE3 (Control Sequence)**
For: 5’ CATCCTATAAGCTTGGCAGGAG – 3’
Rev: 5’ TGCTGGTTAGCAGGACTCGG – 3’
Amplicon: 229 bp
**B4-RE1**
For: 5’ GCACACAGCCTGTTTCTCAACG – 3’
Rev: 5’ CATGCACCGACTAGTCGCCG – 3’
Amplicon: 248 bp
**B4-RE2**
For: 5’ GAGATCATGGCCCAGATAGCAG – 3’
Rev: 5’ GGTCAGAAAGATCAAGTTTGTGTC – 3’
Amplicon: 196 bp
**Control Exon 3 BMP2**
For: 5’ GTGACGTGGGGTGGAATGAC – 3’
Rev: 5’ CAGCATCGAGATAGCACTGAG – 3’
Amplicon: 200 bp
**Control Exon 5 BMP4**
For: 5’ CTGCCGGCGCCACTCGCTC – 3’
Rev: 5’ CAGCATGGAGATGGCACTCAG – 3’
Amplicon: 232 bp
