# Supplementary material for: BMP-Mediated Functional Cooperation between Dlx5;Dlx6 and Msx1;Msx2 during Mammalian Limb Development
Source: PLoS One. 2013 Jan 29;8(1):e51700. doi: 10.1371/journal.pone.0051700 (PMC3558506; doi:10.1371/journal.pone.0051700)
Supplement: Table S3 — The Dlx5 Position-Weight matrix and results of the prediction of Dlx5 binding sites based on genomic conservation. (PDF) [file pone.0051700.s006.pdf]

Table S3

The Dlx5 Position weight matrix and results of the prediction based on conservation

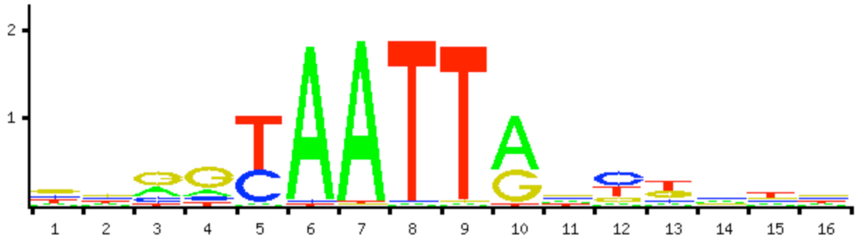

We used the positional weight matrix provided by JASPAR under accession PH0024.1. We also generated our own PWM (below), which is very similar to the one above.

```
A   [11 17 28 18  1 97 98  1  1 61 19  5  7 26 15 19 ]
C   [29 29 17 14 38  2  0  1  0  0 25 49 17 34 22 27 ]
G   [37 37 49 59  0  0  1  0  2 38 37 17 37 23 22 35 ]
T   [22 17  5  9 61  1  1 98 97  1 18 28 39 17 41 19 ]
```

Since the PWM is quite long (16 positions) we used a cut-off equal to 1/2 of the maximum possible score. This produced 565995 sites in the whole mouse genome.
We then selected among the sites identified above, the ones that are conserved in at least one of 8 species. A site is defined conserved with species *S* if it lies in a region of the mouse genome which is aligned with a region of the *S* genome (using the axt alignments provided by UCSC), and the aligned sequence in *S* is a site according to the same definition used for mouse sites.
The number of sites present in the mouse genome and conserved in the genomes of other species is reported below:

genome	conserved sites
hg19	30704
bosTau4	24148
monDom5	10019
ornAna1	6840
galGal3	4663
xenTro2	1890
danRer6	1010
petMar1	76

The conservation score of a site corresponds to the number of species in which it is conserved. The following table reports the number of sites for each value of the conservation score:

score	sites
1	21380
2	11262
3	4085
4	2295
5	1453
6	812
7	254
8	12
