# Supplementary material for: BMP-Mediated Functional Cooperation between Dlx5;Dlx6 and Msx1;Msx2 during Mammalian Limb Development
Source: PLoS One. 2013 Jan 29;8(1):e51700. doi: 10.1371/journal.pone.0051700 (PMC3558506; doi:10.1371/journal.pone.0051700)
Supplement: Table S4 — Sequences of the mouse and human conserved genomic regions containing predicted Dlx5 binding sites near the BMP2 locus. (PDF) [file pone.0051700.s007.pdf]

Table S4

Sequences of the mouse and human conserved genomic regions containing predicted Dlx binding sites near the BMP2 locus

BMP2 locus

Element B2-R1 (30 kb upstream of the Start codon of Bmp2)

Score: 1 **Position:** chr2:133347131-133347146

Band: 2qF2 **Strand:** +

```
>mm9_dna range=chr2:133346631-133347646
GTCAAATTAAGGTATCTAATCTGCCACAGGGACCAGCCAAAATGTCAAAA
GACTCCCAAGCACTCCAGCAGCGAGCCATATCTGCCTAGAAGGAAATAGT
TGCCAATACCTTTAGAAGTAATTGTTTTGATGGAAGATATCACAAAAGCC
TAGTTATGGCTACTTTGCCCACTTCATACTCCACAAATGACTTAGTTTCC
GTTCCCTGAGGCTCCCTGCCAGTCACTCAACAAGCCAACCAGTACACTTA
ACAAGCTGGCCCATCACCATGTTGAAAATATACAACATGCCTTTTCTAAA
CGCTGGGCTGGGAAAAGAAAGATCCCTAGATGCTTTTCCCAACATTTTCC
CTGGCAAATCCAATCTTCACTCGTTTTGTAATTTTAACTCCCAAGGGAAA
ACAGACAGCAGCCGTGTCTCAGAAAGAGCGTCTCCCGTAGAGCACCAGAG
ATGTGGTATTTTAATAGCTGCCTTCAGCCCCTCCCCTGGAAACATATTAT
GTGGTCATTAGTGATGGGCAAACCTCTGTGGGTTTCTAGGTTTGAATCTG
TGCCAGAAGTTCCAAGTTCCTGCTTCTTTTGACctctctctctccctctc
tctctctctctctctctcCCCTTCATTGCTTTTTGTTGTATTTAATTC
CTTCTCACTTAACTATCTAGCCATATGCTTCAATCTCTTCTTCATATAAA
TAAGAAAAGGGAAGAGGTGATGGATAGGATATGTGAGTTCCCCGCTTCTA
TCTCTGTTGACAGCTCATGTGCCAAACAGGATTGTAATCAGGCCCCAATG
AGGCACAGCCAAGAATAAGTGACTAGAAACTCACTGTGTGCCCACAGTCT
AAAGATGCCCAGGGTACTTGTAGGTGAATAAGAAACTCTCCAAAAGATGC
TGAGACCAGTGGGCCAGTAGGACAGCACAAACACAGACCCAGTAGGACCC
TGAGCATGGAGAGGGGTCTAGGATCTGGAAAGCTGGAGAGAATGCTTGGA
ACAGTTGAGAAACCTA
```

B2-R1 in Human *(GRCh37/hg19)* compared to *Mouse (NCBI37/mm9)*

Conservation: 98.3% of bases, 100.0% of span

Position: chr20:6711276-6712353

Band: p12.3 **Strand:** +

```
>hg19_dna range=chr20:6711276-6712353
GTGAAATTAGGGCTTTTCATAGGCCACAGTGATCAGTCAAGATGCCCGTA
GACCCATGTGTACTCCACCCAAGAATCCTATCTTTCTAGAAGGAAAGGCC
GAGTTGCCAACACCCGTTGAAATAACTGTTTTTATTGAAGACATCACAAA
AGGCTGGTTCTGGCTACCTTGGCCACCTCATACCCCACAAATGGTTTTGT
TTCCGCTCACTTACGGCTGCCTGCCAGTCACCCAGTAAGCAAGCCAGCAC
ACTTAACAAGCTGGCCCATCACCACATTTAAAATATACAACGCAAACGCA
GGCATTTCCAAACCCTGTGCTGGGGAAAGAAACCTCCCCAGTGAGTTTTC
CCAACATTTTACCTTTTAGAAGCCAAACTGCAGTCATTTTTCCCTTTGAC
TCTCAAGGGAAATCTGACAGGGACCACGTTTCAGAAAACTTGTCTCCCAT
AGAGCACCAGAGCTGCGGTATTTTAATAGCCTCTCTCAGCCCCTCCCCTG
GGAACATATTATTATGTGATCATTAGTGATGGGCAAACCTCACTGGGTTT
CCAGGTTTGAGTCTGTGCCAAAAGTTCAGAGTTCTTCTTTTCTTGACAAC
TCAAAATCTCCCCTATTTTTCCCTTTTATTTATTCATTTGTTTGTTTATG
TGTGCTTTGCTTGGTTTCTTCCTATCTTACCACACTCTCCAGCCTTTTCT
CCACTTTACACACAGAAGAAAGGAAGGAGATGGGGCAGAAAGCATGAGTT
TCTTGCTGGTTTCTCTCCATTGAGGTGCACTGGATGAAACAGAATTGTAA
TCAGGCCCCAATTACTGGCACAACCAGAAAAGAGTGACTAGACACCTGCT
ATACGCCCAGGACCACAATTCCGGGGGTGCCCAGTGAGTTTGTAGGTGAG
TAAAGGATAAAGGCTTTTCTTAGAAGGGTTCCCATTCCAACTGGGATGCT
GGGACAAACAGGCCAATAGATGTGTTGACGAAGGCATGCAGACATTGCTC
CACCAATATACTTTAAGGACTAAGCAGTCAAGTTTACAGGAAGACTGAAT
GCGGTGCATTAAGAAGTTGTAAAACAAA
```

Element B2-R2 (3.5 kb downstream of the 3'UTR of Bmp2)

Score: 1 **Position:** chr2:133392125-133392140

Band: 2qF2 **Strand:** -

```
>mm9_dna range=chr2:133406066-133407181
cagcagtatggaagtgtaagccgaataaacccctttcctcccaaacttgct
tcttggtcataatgtttgtgcaggaatagaaaccctgactaagacaGAGG
CTGACCTTGGTTTCCCCACAATGTCAGTTCAGGAATAACATAACTGAGGC
CCCGTTTGCATTTCAGAGGTGGGGATGTTGCTCCTCCCAGAAGCAAAGC
TGGCTGCACAGCTGCTCACAGGACTTGTGTCAAGCCGAAGTTCTCTGGGA
CATGCATGGACCAAAGACTATGAGAGCCTTGCTTGGCTGTATCTCTTCTG
TCTCTGGCATCTTATAAGTAAGAAGAGAAAGCTTTGTTTTCAGTTTTCTT
TTCCTTTTTTCCCCAACTAATCTTCTGCCATGAAAAAGACAGTTATACAT
AAACTACCTGGCCCGAAAGGAATCTTCAGCCCTAGGAGCTTGGCAGGGAG
GCATTCAGAAAGCACTTGTAAATCTACAATCTAGTCTTGTGGCCCTGCCA
GGGAACTAATTACCACCTCCAAGAAATAGGTAGAAATTATGAATTCATTT
TCTCCTGGTGAGAGAGAGTGCTGAGGAAGTAAGGAGCCAGGAATATCCTT
CTCTCGGGGACTGAAGGGTCATCTTACCAGCAGAAGGTTCTGGTCCCCAG
CATGCAAGACGAAGGAAAAGGGGTCAGTAAACACTTATCTTATCAACAGC
TGCTAATGGGTAACCACTATTCTCACAAACTCCAAGAGAGCTACTTCACT
GGGGTTTCAGGGTGGGCAAATGCCAGGAGTTACAAGTTTctctccctctc
cctctccctctccctctccctctccctctccctctccctctccctctccc
tctccctctccctctccctctccctctccctctccctctccctctccctc
tccctctccctctccctctccctctccctctccctctccctctccctctc
tctctccctctctctctctctctctctctctctctctctctctctctctc
tctccTGTGTGTGTGTGTGTGTTTTATATATTGTAATTTTATCTCTAGAT
GCTTCTTTCTAGATGACTAGATGGGCTTCAGGAAGAAGTCTATCCAAATT
TCTTCCCTTTCTCTGT
```

B2-R2 in Human *(GRCh37/hg19)* compared to *Mouse (NCBI37/mm9)*

Conservation: 91.7% of bases, 97.4% of span

Position: chr20:6764356-67165307

Band: p12.3 **Strand:** +

```
>hg19_dna range=chr20:6764356-6765307
tgtgtctgtgaatgtatatgtacatatgtaATGATTTGTTCCTATCTGGA
GTCCAAATGCCAAGCATAGGGCACCTTCTTCTGTTTTACTGAGTTTCTCA
TTTTGCGTAGGTCTCTGAATTTCGCATTGAGTCCCTCTGAGGTGCTTAAG
CAGTACTAAGCAAGACACTGACTTTAACACCACTTAAAAGCTTGCGTTTG
CCCCATGTCCTGGGCCCCACTCCAAGGCATTGCCAGAAGTACATTATTGT
TGCAGTCCATTCTGATGAGGAGGTCTCTAAGCAAACCACCGATCTGCTTA
AAGCCTCCCTCATAAGGAGCAGGATCTCATCTGGACTGCATTTACACTGG
AACCGCTTGGCCACAACACTATGAGAGAATAATTATTGAACTATTTTACT
GTACAACTATTTCCAAATTGTTGATTTTTTATTGATTTCAGGAGGCAAGC
TAATTGCCCCATGGCAACTTGATGTTAACATTTAATGAAGATCAGGAAAC
ACAGTTTATGAAAAGCTTTTGTATAGTAACTGCAAAGTTTCAAGAACTGT
TTTATACTTCCTGACAGTGCTGGGCCTAGCTTAATGGTTTGTTTTTATTG
CTTAAGAGTGTAAACCTTGCTTTAGCCCGGTGTGTGGAAACTATACCCTC
ATCCTGCATTACACATTTTCTCATTGCTTTTTACTCACTCCTGACTTCCT
TTCCTGATATCTACAATTGAGACTTTTTCTGTTTTAATTGTCTCAAAATG
AGTTGCTATCTTTGCATGTTGAGAATCTAacacacacacacacacacaca
cacacgtgcacgcatacatacTCATGAAGTTAAGTGTCTGTATTGTTTCA
CTTTGTTTTGGGTGGAGTCTCTTTTCGGCACTTGGTTGCTAGACGACCAA
GCAGACAAAAGTTAAAGATTGAAAAGGCATTTTTAAAGATGCCAAATCTT
```

Element B3-R2 (18 kb downstream of the 3' UTR of Bmp2)

Score: 1 **Position:** chr2:133406566-133406581

Band: 2qF2 **Strand:** -

```
>mm9_dna range=chr2:133391625-133392640
cacacgtacatGCTAACAAGCACACATGTTCACATGCACGCATGCACACA
CATGCATGCACATCTCTGAAGGAATTTTCGTTTGCTCATGTCTAGAGTCT
GAATGCTGAGGCAAGAGCATCAACCGTCCCCCACCCCACCCCCTTGGCCA
ATCCCTATGGATTTATCATTTTGTTTAGGTCTCTAAACTCTGAATGGAGA
CCCTCTGAGGTGCTACACCATACGCTGACTTTAACAGCACTTAAAAGCTT
GTGTTTGCCTGTGTGCTGGGCCCCTCGCCAAGGCATTGCTGGGAGTCCAT
TACTGTTGCAGTCCATTCTGATGAGGAGGTCTCTCAGCAAAGCCCCGATC
TGCTCACAGCTCTCCTCATAAGGAGCAGGAACTCATCTGGACCGAATTTA
CCCTAGAACCGCTTGGCCACAACACTATGAGAGAATAATTATTGAACTAT
TTTACTGTACAACTATTTCCAAATTGTTGATTTTTTATTGATTTCAGGAG
GCAAGCTAATTGCCCCATGGCAACTTGATGTTAACATTTAATGAAGATCA
GGAAGCAGAGTTTATGAAAAGCGTTTGTATAGCGACTGCAAAGCTTCAAG
AACTGTTTTACACTTCCTGACGGGGCTGGGCCTCGCTTAATGGTTTGTTT
TTATTGCTTAAGAGTGTAAACCGTGCCTTAGCCCAGTGTGTGGAAACTAT
ACCCTCATCCCGCATTGCACATTTTCTTGTTGCTTTTTCCTCGCTTCCCA
TGTCATTGTACCATCTACAATCAAGACTTTCTCTTCTTTGTATGCAAGCA
AGTTTCTATCTTAGACGTTGATGACACTacatgcacacacacacacacac
acacacacacacacacacacgcacatgcatgcacaTAGCCTGATGAACTG
AATGTGCTGCTTCACTGTGTTTGGGTGGAGTTTCTGTTCAGCACTTGGTT
GCTAGACAACCAAGCAGACAAAATTTAGCGATTCAAAAGGCATTTTTAAA
GACGACAAATTTTGAG
```

B2-R3 in Human *(GRCh37/hg19)* compared to *Mouse (NCBI37/mm9)*

Conservation: 68.1% of bases, 90.7% of span

Position: chr20:6780656-6781511

Band: p12.3 **Strand:** +

```
>hg19_dna range=chr20:6780656-6781511
CCCTGGTCTTCCCACACAGCTCAGCACAAGAAAAATGTTTCTTTGAAGGA
AGCAGCCTCTAGGACCTATGACAATTTTCAAGATTTTTTTTTTTTCTAAG
TTGCATTCTCTCCGCTACAGAAGTGGCTCAGTTGATGCTTTGGAAGTTGT
CTCAGCTGGTTCAAAAAGCTCCTATGTTACCTTCATTTTCAGGGACAGCA
ATGAGCCTGACTCTCCTGCATCTCCTTTGTCTGAGGCATAGACCACTGAC
TGCTTATGGAAAAGAACAGATAATGATATCCGTCTCCTGCTTCCACCCAC
CACTCAATGTAACTTTCTGCCATGAACATAACCAGCCACACATAAACTGT
CTGCAGAAAAGGAAGTTCCATCCTATAAGCTTGGCAGGAGGGTATGTAGG
GTCAACAAAGAAATCTCTGCTACTCGTTTGCAGTCATTTCCATGAGGCTA
ATTGTCACCTCCAGAAATCAGCCCAGAAGGAATAAATGCATTTTCCCCTG
ATAAAAGAAAGGACCTAAGGAAATGGGGATTGCAGATTAGCCCTTTCACA
GGAACTGACTGGGCCTGTAACAGCCATCCGAGTCCTGCTAACCAGCAGAT
AAACTAGATAAGAGTTTACATTAGCTTTGGCTGCTGTGAGGATGCCTCTG
CTCTCTTGGACTCCAGAACAGTTGGTTCATTAAAGTACCAGGGTCGGCAA
GTGCCAGGGTCATAAGTCTGAGTATTTATGTTTTATAATCCTCCATTCGA
TGTTCTTTTTTGTATGTGTCTAGAGATAACGGTTTCTCTCTAGATCCATC
CTTCTAGGTAACTGTATGGCATTCAGGAAGGCATCTGTACAATTTCTCCT
CTCTTC
```
