# Supplementary material for: BMP-Mediated Functional Cooperation between Dlx5;Dlx6 and Msx1;Msx2 during Mammalian Limb Development
Source: PLoS One. 2013 Jan 29;8(1):e51700. doi: 10.1371/journal.pone.0051700 (PMC3558506; doi:10.1371/journal.pone.0051700)
Supplement: Table S5 — Sequences of the mouse and human conserved genomic regions containing predicted Dlx5 binding sites near the BMP4 locus. (PDF) [file pone.0051700.s008.pdf]

Table S5

Sequences of the mouse and human conserved genomic regions containing predicted Dlx binding sites near the BMP4 locus

BMP4 locus

Element B4-R1 (proximal *BMP4* promoter)

Score: 1 **Position:** chr14:47010669-47010684

Band: 14qC1 **Strand:** +

```
>mm9_dna range=chr14:47010169-47011184
CTCTCAGCTCGGATGCCGAACTCACCTAGCTTCCGGGCCAGGCCTCGCGc
tcttgccttcccaccctccagcaccaccctctcacatctactccttcctt
ctcttttccctccctctctttcttcccttcctccccagtctttcccccca
gcggcccctcccttcttccctcctcccttaatcgcctccccttctGGGGA
TGGGAGCCCCGCCCACATCCTCCCATCCAGCCACGGGGCCTCGCCTCGCC
TGGCAAGGCCCGGGTCCGGAAGATTGCACAGCTCCCCAGCCCTCAGTTGC
CACCTTGGCTGGGACCGATGCCTCCAGCTCGGGAAGCAGCCGGGGCTCAC
CTGGGGACCACGTGCAAGAGGTACTAGAAAGCATGCACCAACTAGTCTCT
GTACCTTCCAAAAATACCCATGGGAGTCTGGGCTTCCCTGAGTTTAGAAT
AACTGCTGCCCAAACTGATGATTAAAACACTGAGTAATCACCATTTACCC
CGAGTAATTAGAGATAATGAAACACCTCTAAAATCAGGTTATGGAGAAGG
GAGCTGTTGGATTGGTTTAAAGGGTGGCCTTCCGTAAACTGTTAAAGGAT
TCCCAAACGTTGAAACAGGCTGTGAGCAGGGCTGTGTGCCCGAAGGAGAT
ACCTCTTCAACTATTAGGAGGTGACTTTCCCCCCAAAAAAGTTTTTGTGA
GATGTTTCTTGTGGACATTTGAGAATTGCACCAACCCCCCTTAAACTGGG
TGCCCTCCATCGATCCCCCAGGTCTAACATTTCAAGTGCACAGAGGTAGT
TTGCAGGCACTTGGCAGGCAAACAGGCCTAGGCCTTGTAAGACAATTAGA
AAATCCTATTTATATCTCTGAAGCCTTCTTAACTGCCAGATGGTTATttt
gtttgtttgtttgtttgtttgGGACCTTCCAGGAAGGTAACACAGTGCCT
TTTAGGCCATGTAGAGAAAAAAACAAAGTGCCCTTTGGCCCCTTTTTAAA
AGAAGGGAGATGAGGA
```

B4-R1 in Human *(GRCh37/hg19)* compared to *Mouse (NCBI37/mm9)*

Conservation: 96.2% of bases, 100.0% of span

Position: chr14:54423452-54424504

Band: q22.2 **Strand:** +

```
>hg19_dna range=chr14:54423452-54424504
CCCTCAGCTCGGATGCCACACTCACCTAGCTTCCGGGCCGGGCTCCGCGc
tccttccctccctccctcctcctctgccttctcgcatcttccttcctcct
ctttccctccctcgctttctttctttccttcctcctcctcttccccggcg
gcccctccctccttccctcctccctcgctcgcctccctTTCTGGGGATGG
GAGCCCCGCCCACATCCTCCCCTCCAGCCGCCCGACCTCGCCTAGCAGGC
CCGGGTCCCGGAAGCCCAGGCAGCGCCCGAGTCCGCAGCTGCCGTCGG
CTGGGACGCCGCTGCCTCCAGCTCTGGGAAGCGGCTGGGGCTCACCTGGG
GACCACGTGCGGAGGTACTAGAAAGCATGCACCGACTAGTCGCCGTACCT
TCCAAAAATACCCATGGGAGTCTGGGCTTCCCTGAGTTTAGTGTAACTGC
TGCCCAAACTGATGATTAAAACACTGAGTAATCACCATTTACCCTGAGTA
ATTAGAGATAATGAAACACCTCTAAAATCAGGTTATGGAGAAAGGAGCTG
TTGGATCGGTTTAAAAGGTGGCCTTCCATAAACTGTTAAAAGATTTCCAA
ACGTTGAGAAACAGGCTGTGTGCAGAACTGTGTGCCAGAGAGAGATACCT
CTTGGGCTGTTAAAAGGTGAATTTCCTTCCAAAACATTCTTAAAGTGCGT
TTTGTAGAACACCTTGGTGATGACCCTGAGGTAGACCCCAGTAAATGAGC
TCACTTCCCTCCTCCCCCCAAGGCCTAAAGACAGAGGTGGCCTGCAGACA
GGCTGGGGCCACGTTTTTTACAGTTAGAAAAATCTGTTTCTAGCTCAGAA
GCCGTCTTAAGAACCGACACAGCAgttatttttgttggtgttatggttgt
tgttgatttttttgttCATTTTTGGTGTACCTAAGAGTCCTGACACTCTG
TCTTTCCAAGGAGATAACCACAGAAGCAGTAACCTTTTAGGCCACAGGGA
GACAAGAAAAGATGCCCCTTGGTCTGTTTTTAAAGGAAGAGGGGAGATGA
GGA
```

Element B4-R2 (intronic)

Score: 1 **Position:** chr14:47005978-47005993

Band: 14qC1 **Strand:** -

```
>mm9_dna range=chr14:47005478-47006493
CTCCTCCTCCCCAGACTGGAGCCGGTAAAGATCCCTCATGTAATCCGGAA
TGACGGCGCTCTTGCTAGGCTGCGGACGGCGGCGCAGCCCAAACATCTGT
AGAAGTGTCGCCTCGAAGTCCCGCAGGAGCTCATGGCTCTGCCCTGAGCG
GCGTCCTCCCGCGTGGCCCTGAATCTCGGCGACTTTTTTCTTCCCGGTCT
CAGGTATCAAACTAGCATGGCTCGCGCCTCCTAGCAGGACTTGGCATAAT
AAAACGACCATCAGCATTCGGTTACCAGGAATCATGGTGTCTCTGGGGAG
GGGGTTGGGGTGGAAGGTTAAAGAATAAATAAACACCGATCAATAGCGAG
AAATAGAGATGTCTCTGCATATGCATATAGGGGTAGCAACCGACGGTCAA
GATGTAAAACAGGTCCGAAAGATCAAGTTTGTGTTTTCTCCCTCACACCC
TACACACACACACACCAGCTGCAGCCATGCACGGCCCGAAAGTAAGAATC
GCCCTGTAATTACTTGGTCTAACTTGTTTACACTCAAATAACCCCAAATC
AGATAGCCTCCATCCTGTCCCAACGGCTATCTGAGCCATGATCTCAGCGT
GGCTTCTTCAAGGATAAACAGTTAACATTGAGGAGGTAAAGGGGGGTGGG
GGAGGGAGAATTAAAATGCCATCGCTCTTCACTTCGGaccttcagcctcc
cccccaccttctccacccccccccccacccccgcccttcctccgccctcca
gcccaatttccacAACTTCCAGCTGGTTAAGAACAGGAGGAGGGGAGAAC
AGAGCTGTGCCGGCAGGGCTTGGACCGGGCAGCAAGGTCCAGTGACTGGG
AAAAGGAGTCAGGGGGCTGCCCCAGTGACTTTGTGATTTATTTCATTGTA
GTCGCTGCGTTGAAAATGCTCACTAAAGAGTCTTGTTCCCAGGGAACGGT
AGGGGTGATTCCAAAACGAACATGGttttgttttttttggtttttttttt
tttttttttATGAGGC
```

B4-R2 in Human *(GRCh37/hg19)* compared to *Mouse (NCBI37/mm9)*

Conservation: 94.6% of bases, 100.0% of span

Position: chr14:54418656-54419651

Band: q22.2 **Strand:** +

```
>hg19_dna range=chr14:54418656-54419651
CTCCTCCTCCCCAGACTGAAGCCGGTAAAGATCCCGCATGTAGTCCGGAA
TGACGGCACTCTTGCTAGGCTGCGGGCGGCGGCGCAGCCCAAACATCTGC
AGAAGTGTCGCCTCGAAGTCCCGCAGGAGCTCATGGCTCTGCCCTGAGCG
GCGTCCTCCCGCGTGGCCCTGAATCTCGGCGACTTTTTTCTTCCCCGTCT
CAGGTATCAAACTAGCATGGCTCGCGCCTCCTAGCAGGACTTGGCATAAT
AAAACGACCATCAGCATTCGGTTACCAGGAATCATGGTGTCTCTGGGGAG
GGGGAGGGGAGTGGAAGGTTAAAGAATAAATAAACACCAATAAATAGGGA
GAAATAGAGATGTGTCTGCATATGCATTTAGGGCTAGAAATGGAGGGGCA
AGATGGAAAGCAGGTCAGAAAGATCAAGTTTGTGTCTTCTCCCTCACACC
ACCCGCCCACCCACCAGCTGCAGCCATGCACGGCCTGAAAGTAAGAATTG
CCCTGTAATTACTTGGTCTAACTTGTTTACAGTCAAATAACCCCAAATCA
GATAGCCTCCATCCTGTTAATCTTTTTTTGTCCTAACTGCTATCTGGGCC
ATGATCTCAGCGTGGCTTCTTGAAGGATAAACAGTTAACATTGAGGGGGT
GAAAAGGGGTGGGGGAGGGAGAATTAAAATGGCATTGCTCTCCACTTTAG
ACCTTCAGCTTCTCTCCCTTTTCCTCCACCCTCAGAGCCCAACTTTCACA
ACTTCCAGTTGGTTCAGAGCCAGAGGGAGGGAAAGTGAGAGGCAGGGGCT
GGGCGAAGACAGAGGGCGACAGGGTCCAGAAGCCGAGAAGGTGGTCTGGG
AGCTAACCGGGTGGCTTGAGGGCTTATTTTATTGTGGCCGATGAGTTTGA
AATATTCAGCCAAAAGTGCCTGATCGTGCCCGCTCATCCCCAGGGAAGGG
GTGAGAGACGATTCCGGAGAGACCTCTGTGAGTTTTGACAAGAGAG
```
